# Layered Double Hydroxides: Recent Progress and Promising Perspectives Toward Biomedical Applications

**DOI:** 10.1002/advs.202306035

**Published:** 2024-03-19

**Authors:** Lei Li, Irem Soyhan, Eliza Warszawik, Patrick van Rijn

**Affiliations:** ^1^ Department of Biomedical Engineering University of Groningen University Medical Center Groningen A. Deusinglaan 1 Groningen, AV 9713 The Netherlands; ^2^ W. J. Kolff Institute for Biomedical Engineering and Materials Science University of Groningen University Medical Center Groningen A. Deusinglaan 1 Groningen, AV 9713 The Netherlands

**Keywords:** biomedical application, drug delivery, exchangeability, layered double hydroxides, nanotheranostics

## Abstract

Layered double hydroxides (LDHs) have been widely studied for biomedical applications due to their excellent properties, such as good biocompatibility, degradability, interlayer ion exchangeability, high loading capacity, pH‐responsive release, and large specific surface area. Furthermore, the flexibility in the structural composition and ease of surface modification of LDHs makes it possible to develop specifically functionalized LDHs to meet the needs of different applications. In this review, the recent advances of LDHs for biomedical applications, which include LDH‐based drug delivery systems, LDHs for cancer diagnosis and therapy, tissue engineering, coatings, functional membranes, and biosensors, are comprehensively discussed. From these various biomedical research fields, it can be seen that there is great potential and possibility for the use of LDHs in biomedical applications. However, at the same time, it must be recognized that the actual clinical translation of LDHs is still very limited. Therefore, the current limitations of related research on LDHs are discussed by combining limited examples of actual clinical translation with requirements for clinical translation of biomaterials. Finally, an outlook on future research related to LDHs is provided.

## Graphical abstract

Layered double hydroxides (LDHs), recognized for their biocompatibility and unique properties, are extensively examined in biomedical contexts. This review delves into recent LDH advances spanning drug delivery, cancer diagnosis and therapy, tissue engineering, coatings, membranes, and biosensors. Despite the significant potential, clinical translation remains limited, prompting a discussion of current research constraints and an outlook on future LDH‐related biomedical investigations.

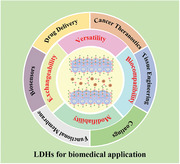

## 1 Introduction

**Figure 1:**
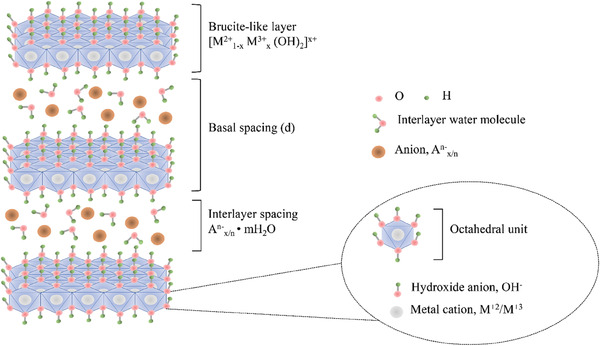
Schematic diagram of LDHs structure.

2D‐structured nanomaterials have gained significant attention from the scientific community due to their unique physicochemical properties, including a high surface‐to‐volume ratio, surface functionalization, distinct shape and variable nano‐length sizes, adjustable mechanical properties, and high anisotropy, making them attractive for various biomedical applications.^[^ ^1^ ^]^ Recently, LDHs have emerged as promising 2D nanomaterials and have been extensively studied for biomedical applications.^[^ ^2^, ^3^ ^]^ The initial LDH was discovered by a German‐Austrian geologist, Hochestetter, in a naturally occurring mineral in 1842.^[^ ^4^ ^]^ Due to its resemblance to talcite and its water content (hydro), it was named as hydrotalcite. The composition of hydrotalcite is Mg_6_Al_12_(OH)_16_CO_3_·4H_2_O, and its structure is similar to that of brucite Mg(OH)_2_.^[^ ^5^ ^]^ With the development of modern analytical and characterization techniques, researchers determined the layered structure of hydrotalcite and successfully synthesized it artificially.^[^ ^6^ ^]^ With a deeper understanding of the structure and composition of hydrotalcite, researchers realized that the type and ratio of metal ions and the type of interlayer anions in the hydrotalcite structure could be adjusted.^[^ ^7^ ^]^ As a result, researchers synthesized a series of hydrotalcite‐like compounds with compositions different from the original hydrotalcite.^[^ ^8^ ^]^ Natural hydrotalcite, artificially synthesized hydrotalcite, and the subsequent series of hydrotalcite‐like compounds are collectively referred to as LDHs. As shown in **Figure** **1**, LDHs have a layered structure of double hydroxides with two types of metal ions, M(II) and M(III). Metal ions and water molecules are arranged in hexagonal layers in a certain proportion in the crystal lattice. The general chemical formula of LDHs is [M(II)_1‐x_M(III)_x_(OH)_2_]^x+^[A^n−^]_x/n_·mH_2_O, where M(II) (e.g., Mg^2+^, Zn^2+^, Cu^2+^, Ni^2+^, Co^2+^, Fe^2+^, Mn^2+^, or Ca^2+^) and M(III) (e.g., Al^3+^, Fe^3+^, Co^3+^, Mn^3+^, Cr^3+^, Ga^3+^, Gd^3+^, or In^3+^) represent divalent and trivalent metal ions located in the host layers, A^n−^ represents the interlayer anions (e.g., CO_3_^2−^, NO_3_^−^, Cl^−^, etc.), n represents the charge of the interlayer anion, m represents the number of water molecules, and x is determined by the molar ratio of M^3+^/(M^2+^ + M^3+^).^[^ ^9^, ^10^, ^11^ ^]^ Compared with the fixed composition of the natural mineral hydrotalcite, the metal ions in the later synthesized LDHs have higher variability and can be replaced by ion exchange and other methods, forming compounds with different functions. In addition, LDHs have good controllability, and their physical and chemical properties can be controlled by adjusting parameters such as the proportion of metal ions, the type of interlayer anions, and the water content, making them suitable for various functions such as catalysis,^[^ ^12^ ^]^ adsorption,^[^ ^13^ ^]^ separation,^[^ ^14^ ^]^ and drug delivery.^[^ ^15^ ^]^

**Figure 2:**
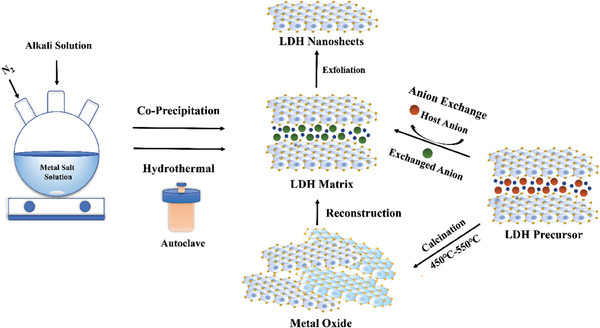
Schematic diagram of common synthetic methods for LDHs.

Synthesis methods of LDHs mainly include direct synthesis and indirect synthesis, among which co‐precipitation and hydrothermal synthesis are commonly used methods of direct synthesis of LDHs. The co‐precipitation method is widely used in the production of industrial LDH products and related research due to its simplicity and mild reaction conditions.^[^ ^16^, ^17^, ^18^ ^]^ Specifically, as shown in **Figure** **2**, LDHs can be obtained by slowly adding an alkaline solution to the metal salt solution while adjusting the pH of the co‐precipitation system under continuous stirring, followed by washing and drying.^[^ ^19^ ^]^ Notably, the target anions can also be introduced into the co‐precipitation process to achieve one‐step loading of target anions onto LDHs. Due to the simplicity and efficiency of co‐precipitation, it is also widely used for one‐step drug loading of LDHs in biomedical research.^[^ ^20^, ^21^, ^22^, ^23^, ^24^, ^25^ ^]^ However, the co‐precipitation synthesis method of LDHs has some drawbacks, such as low crystallinity of the precipitate and a broad particle size distribution.^[^ ^18^, ^26^, ^27^, ^28^ ^]^ Compared to the co‐precipitation method, the hydrothermal synthesis method has more stringent requirements for the temperature, pressure, and reaction time of the LDHs synthesis process, which involves adding the mixture of alkaline metal salts to the hydrothermal reaction vessel to promote the hydrothermal reaction of ions to generate LDHs.^[^ ^18^, ^29^, ^30^ ^]^ Although the reaction conditions of hydrothermal synthesis are more stringent, and the process is slower and more tedious, it can better control the morphology and size of LDH crystals, which may make LDHs meet the needs of more complicated applications.^[^ ^31^, ^32^, ^33^, ^34^ ^]^ In recent works on LDHs used in biosensors, the hydrothermal synthesis method is frequently used as it can reasonably control the morphology and size of LDHs, ensuring the accuracy and sensitivity of LDHs‐based biosensors.^[^ ^35^, ^36^, ^37^ ^]^

In some instances, it can be challenging to directly synthesize LDHs intercalated with target anions using the direct synthesis method. Furthermore, there may be issues with low efficiency in loading the target anions onto LDHs, which can subsequently impact the application effectiveness of LDHs. Therefore, based on the interlayer anion exchange properties of LDHs, the ion exchange method has been widely used for LDHs.^[^ ^38^, ^39^, ^40^ ^]^ To prepare LDHs loaded with target anions using the ion exchange method, it is necessary to first prepare easily synthesized LDH precursors through direct synthesis methods, and then further replace the target anions into the interlayer of LDHs through ion exchange. In addition, the “memory effect” of LDHs can also be used to indirectly synthesize target LDHs.^[^ ^41^, ^42^ ^]^ The so‐called “memory effect” of LDHs means that by calcining the LDHs precursor at 450–550 °C, it can be transformed into a mixed metal oxide state. Interestingly, when the metal oxide is placed in the solution containing the target anions, the layered structure of LDHs can be reconstructed and the target anions can be loaded in its interlayers.^[^ ^43^, ^44^ ^]^ However, there are problems with poor crystallinity and low loading rate in the reconstruction method for synthesizing LDHs, and recent studies have rarely used this method.^[^ ^44^, ^45^ ^]^ It is worth noting that the aforementioned preparation methods of the LDHs inevitably result in the generation of byproducts, which in turn necessitates a significant consumption of water resources for their removal.^[^ ^46^, ^47^ ^]^ Addressing this concern, Song et al.^[^ ^48^ ^]^ have successfully devised a green atom‐economic method for the synthesis of LDHs. This approach involves the precise control of the input ratios of metal hydroxides and acidic aqueous solutions during the synthesis process of LDHs, along with the manipulation of reaction time and temperature. Consequently, all components within the raw materials participate in the reactions to yield the desired product or water molecules, thereby eliminating the formation of any byproducts throughout the process, which not only leads to substantial conservation of water resources but also ensures environmental preservation. Recently, LDH nanosheets have also received extensive attention.^[^ ^49^, ^50^, ^51^ ^]^ LDH nanosheets have a larger specific surface area, which makes them more stable, reactive, catalytically active, and easier to modify.^[^ ^52^, ^53^ ^]^ As an illustration, Gu and colleagues have designed an ultra‐thin and colloidally stable nanosheet characterized by an exceptionally high doxorubicin loading capacity of 734%.^[^ ^54^ ^]^ In this nanosheet, doxorubicin maintains stability under physiological pH conditions, while exhibiting controlled release behavior within the acidic tumor microenvironment and lysosomes. This property enhances its therapeutic efficacy against tumors while minimizing systemic toxicity. Typically, the common preparation strategies for LDH nanosheets involve introducing long‐chain anions into the interlayer of LDHs using chemical methods^[^ ^49^, ^55^, ^56^, ^57^ ^]^ or using physical forces generated by mechanical shearing and ultrasonication^[^ ^58^, ^59^, ^60^ ^]^ to exfoliate the bulk LDHs into mono‐layer or few‐layer nanosheets.

As mentioned above, in general, the synthesis of LDHs is not complicated, and its main raw material, metal salts, is inexpensive and abundant in reserves. Additionally, LDHs have many attractive characteristics as biomedical materials, which have led to their widespread application in biomedical research. First, LDHs have good biocompatibility and low toxicity. Although different studies have synthesized LDHs with different compositions and sizes, LDHs have demonstrated safe and satisfactory biocompatibility.^[^ ^39^, ^61^, ^62^, ^63^ ^]^ Notably, the differences in composition and size have little effect on the biocompatibility of LDHs,^[^ ^64^, ^65^ ^]^ which provides a guarantee for their safe application as biomedical materials. Second, LDHs have characteristics such as interlayer ion exchangeability,^[^ ^38^, ^39^ ^]^ high specific surface area,^[^ ^66^, ^67^ ^]^ and adjustable interlayer spacing,^[^ ^68^ ^]^ which make them efficient carriers for drug molecules,^[^ ^69^ ^]^ genes,^[^ ^70^, ^71^ ^]^ and bioactive molecules,^[^ ^70^, ^72^, ^73^ ^]^ with the ability to achieve ultra‐high loading capacity.^[^ ^15^, ^74^ ^]^ Moreover, LDHs as carriers can also provide good protection for the drug molecules they carry, achieving the controlled release effect and effectively avoiding the resistance and side effects caused by repeated administration.^[^ ^75^ ^]^ In addition, as drug carriers, LDHs have the characteristic of pH‐responsive release. Specifically, LDH drug carriers get the drug molecules released very little at neutral conditions, but under mildly acidic environments, based on the high solubility, protonation, and ion exchange of the drug molecules, they can release the drug molecules quickly, achieving the efficient targeted release of the drug molecules.^[^ ^76^, ^77^, ^78^ ^]^ As is well known, the tumor microenvironment is weakly acidic,^[^ ^79^ ^]^ so LDHs can serve as an ideal drug carrier that can rapidly release drugs in the tumor microenvironment but minimally release them in healthy tissues, which may enhance the therapeutic efficacy of anticancer drugs and reduce their side effects. Therefore, LDHs have been extensively studied as drug carriers for cancer therapy.^[^ ^39^, ^72^ ^]^ Additionally, the layer plates of LDHs possess positive charges, which makes it easy to modify LDHs through simple charge adsorption to improve their stability in vivo or perform functional modifications to meet different biomedical applications' needs.^[^ ^80^, ^81^ ^]^ Moreover, because the cell membranes and bacterial films both possess negative charges,^[^ ^82^ ^]^ LDHs with positive charges are more likely to bind to them, thereby improving the binding of LDHs drug carries to cell membranes and bacterial films, and thus improving the efficiency of drug delivery and antibacterial effects.^[^ ^83^, ^84^, ^85^ ^]^ Above all, the ideal biomedical materials should meet complex biomedical application needs, and the flexibility and adjustability of LDHs' structure and composition can enable them to meet various biomedical application challenges. Moreover, the abundant hydroxyl groups on the surface of LDHs and the layer plates with positive charges make it easy to construct multifunctional composites through chemical modification or charge adsorption with other biomaterials.^[^ ^86^, ^87^ ^]^

With a better understanding of the structure and properties of LDHs, leading to remarkable new results, a comprehensive and in‐depth summary of the recent research on LDHs in various biomedical fields will help grasp the latest developments and biomedical‐related research frontiers of LDHs, and further expand the application scope of LDHs in the biomedical field. Although there have been some review articles on the biomedical applications of LDHs recently, most of them only focus on a specific application area of LDHs, such as the application of LDHs in anticorrosion coatings,^[^ ^88^, ^89^ ^]^ cancer treatment,^[^ ^90^ ^]^ drug delivery,^[^ ^69^, ^91^ ^]^ and tissue engineering.^[^ ^92^, ^93^, ^94^ ^]^ Bringing all the progress within the biomedical field together will help identify the commonalities of LDHs in different application areas. In addition, it is worth noting that there have been some review articles summarizing the research of LDHs in different biomedical application areas. For example, Hu et al. summarized the application of LDH‐based nanobiomaterials.^[^ ^48^ ^]^ Hu et al. elaborated on the classification, characteristics, and preparation methods of LDHs in detail, therefore we will not elaborate on these related contents. Two other review articles on the biomedical applications of LDHs by Kankala^[^ ^95^ ^]^ and Pavlovic et al.,^[^ ^96^ ^]^ respectively, mainly elaborated on the construction methods of LDH composites and surface modification strategies in great detail, and for those details, the reader is referred to those works.

In this review, we aim to provide a comprehensive summary of recent advances and achievements in the biomedical applications of LDHs. Drug delivery, cancer diagnosis, and therapy remain the focus of LDH‐related research, and there have been numerous innovative findings in these areas, making a scientific and clear analysis and summary necessary. In drug delivery applications of LDHs, we pay extra attention to LDHs as carriers for non‐anionic drugs, which has not been reviewed so far. In cancer diagnosis and therapy‐related research, the study has gradually transitioned from monotherapy to synergistic therapy and a more nanotheranostics platform, and recent applications of LDHs in imaging are closely integrated with those as well. As a result, a cumbersome and scattered classification of LDHs in cancer diagnosis and therapy‐related research is no longer appropriate, and cannot grasp the development trends of this field. Therefore, for the application of LDHs in cancer diagnosis and therapy, we have provided a concise classification based on current research trends. Meanwhile, we have also conducted a detailed analysis of recent research progress in other biomedical application fields of LDHs, including tissue engineering, coatings, functional membranes, and biosensors. Notably, despite numerous recent advances in LDHs' biomedical applications, their actual clinical translation is still very limited. Therefore, in this review, we not only provide examples of successful clinical translational applications of LDHs but also provide a detailed analysis and discussion of the challenges and difficulties in their clinical translation. Finally, based on the numerous recent biomedical research advancements of LDHs and the challenges in their clinical translational application, we identify the opportunities, key areas, and difficulties for future LDH research.

## 2 LDHs for Biomedical Applications

### 2.1 LDHs‐Based Drug Delivery Systems

#### 2.1.1 Mechanism Study of LDHs‐Based Drug Delivery Systems

Although LDHs have been known for over 100 years and have been widely used as drug delivery systems for decades, there is still a lack of understanding about the drug loading and release mechanisms of LDHs‐based drug carriers, as well as their impact on the immune system, which limits their further clinical translation. Therefore, it remains essential to continue studying the drug loading, release, and immune response mechanisms related to LDHs‐based drug delivery systems to deepen our understanding and progress their clinical translation.

**Figure 3:**
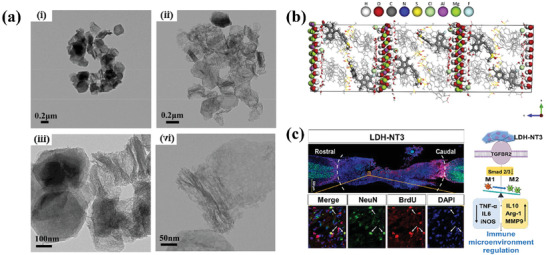
a) Morphologies under TEM of MgAl‐NO_3_ L‐LDH‐MO after released in (i) PBS pH6.5, and (ii) PBS pH 5.2. Layered structures of MgAl‐NO_3_ L‐LDH‐MO under TEM after being released in (iii) PBS pH 6.5, and (iv) PBS pH 5.2. Reprinted with permission from Ref.[99] Copyright 2023, Wiley‐VCH. b) Final model of LDH‐Sulindac after molecular dynamics production run, view along a‐axis. Reprinted with permission from Ref.[101] Copyright 2020, Elsevier. c) Immunomodulatory layered double hydroxide nanoparticles enable neurogenesis by targeting transforming growth factor‐β receptor 2. Reprinted with permission from Ref.[102] Copyright 2021, American Chemical Society.

In previous studies, the pH‐responsive release of LDHs under mildly acidic conditions was attributed to the partial dissolution of LDH structures.^[^ ^97^, ^98^ ^]^ However, these studies only characterized the release behaviors of the drug carriers and lacked relevant data to support the dissolution mechanism. In a recent study,^[^ ^99^ ^]^ post‐release particles were analyzed and characterized in‐depth, revealing that the long‐believed dissolution mechanism of LDHs at lower pH is not accurate. Specifically, LDHs were observed to remain hexagonally shaped and intact at lower pH (**Figure** **3**a(i),(ii)). In this study, the pH‐responsive release behavior was attributed to the pH‐dependent solubility of a drug mimic, methyl orange (MO). MO molecules dissolved faster at lower pH, facilitating the diffusion and release of MO through ion exchange with higher efficiency. However, leakage of Mg^2+^ from MgAl‐LDHs was detected in the release media, which may cause the collapse of the layered structures of MgAl‐LDHs (Figure 3a(iii),(iv)), resulting in incomplete release of the intercalated MO ions. Previous studies have attributed partial release to the formation of phosphate ion grafting on LDH layers.^[^ ^100^ ^]^ Therefore, the results of this study may help to improve and refine our understanding of the mechanisms underlying the partial release phenomenon of LDHs under certain conditions.

Although LDHs have been widely studied as drug carriers, there is limited understanding of the arrangement of intercalated molecules. A recent study employed molecular dynamics simulations to obtain the alignment of sulindac anions in the interlayers of LDHs.^[^ ^101^ ^]^ The results showed that sulindac anions are distributed in the interlayer of Mg_2_Al‐LDHs in a bilayer‐tilted orientation, while water molecules and chloride ions in the interlayer are adsorbed on the surface of LDH (Figure 3b). The X‐ray diffraction pattern of the structural model is in good agreement with the actual experimental results, demonstrating the feasibility and correctness of the calculated structural model. This study provides new insights into a better understanding of LDHs. The quantity and arrangement of ions in the interlayer of LDHs can affect its loading and release process. The combination of molecular dynamics simulation and experimental data can improve our understanding of the loading and release mechanisms of LDHs, and enable reasonable predictions for its release.

Additionally, Zhu et al.^[^ ^102^ ^]^ investigated the neuroregenerative and immunomodulatory functions of Mg/Al‐LDH nanoparticles using a thoracic spinal cord transection mouse model and elucidated their immune‐related mechanisms. Based on RNA‐seq and Ingenuity Pathway Analysis, Transforming Growth Factor‐β Receptor 2 (TGFBR2) emerged as a pivotal gene and target for LDH, which inhibits inflammation and accelerates neural regeneration. As illustrated in Figure 3c, with targeted activation of TGFBR2, LDH downregulates Smad2/3 expression, enhances TNF‐α expression, and increases IL10 expression, creating an appropriate immune microenvironment favorable to the proliferation and functional differentiation of endogenous neural stem cells, thus promoting neurogenesis. Moreover, with the loading of neurotrophic factor 3 (NT3), LDH‐NT3 further expedites axonal growth and neuronal synaptic transmission. Collectively, this study not only advances the development of an immunomodulatory LDH formulation with promising implications for spinal cord injury recovery but also provides invaluable insights into the broader immune‐related mechanisms governed by LDHs, which extend the scope of potential LDH applications in various biological systems.

#### 2.1.2 Functionalized LDHs‐Based Bio‐Nanocomposites

Previous research has highlighted the immense potential of LDHs as drug‐delivery systems. Recent studies have been devoted to the development of functionalized LDH‐based bionanocomposites for the treatment of specific diseases, thereby offering crucial insights for the advancement of LDHs in practical biomedical applications. Most recently, Wang et al.^[^ ^103^ ^]^ reported the development of a composite material, denoted as AFGd‐LDH, which incorporates atorvastatin (a neuroprotective drug) and ferritin (a blood–brain barrier transport agent). The primary objective of this composite material was to mitigate cerebral ischemia‐reperfusion injury (CIRI). Notably, this system exhibits a high capacity for capturing ROS and possesses the capability to traverse the blood‐brain barrier. Furthermore, it enables in‐vivo visualization through MRI imaging, thereby establishing a promising avenue for neuroprotection in the context of CIRI.

**Figure 4:**
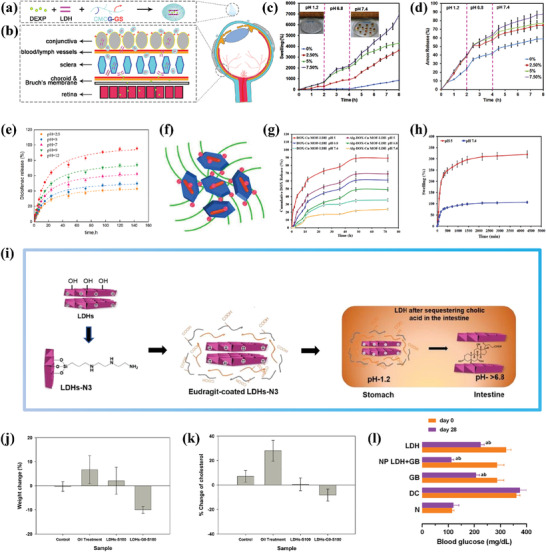
a) Schematic design of multifunctional CMCG‐GS‐DEXP‐LDH. b) The scheme of drug diffusion routes into the retina for the CMCG‐GS‐DEXP‐LDH hybrid nanocomposite eye drop. Reprinted with the permission from Ref.[104] Copyright 2020, Elsevier. c) Swelling profile of CMC/LDH(Cu/Al) bio‐nanocomposite hydrogel beads with different content of LDH in simulated gastrointestinal tract conditions and a digital photo of hydrogel bead in the dry and swelled state. d) AMX release behavior of CMC‐based hydrogel beads with different content of LDH(Cu/Al) wt.% in the simulated conditions with gastrointestinal tract passage (time and pH). Reprinted with the permission from Ref.[105] Copyright 2020, Elsevier. e) Release kinetics of diclofenac from Alg/LDH‐Dic beads at different pHs of release medium. Reprinted with the permission from Ref.[111] Copyright 2021, Elsevier. f) Schematic of DOX‐Cu MOF‐LDH. g) In vitro release profile of DOX from DOX‐Cu MOF‐LDH and Alg‐DOX‐Cu MOF‐LDH beads at different pH values (pH 5, 6.8, and 7.4). h) Swelling ratio of Alg‐DOX‐Cu MOF‐LDH at both pH 5 and 7.4. Reprinted with the permission from Ref.[112] Copyright 2023, Elsevier. i) Schematic illustrating the fabrication process of surface functionalized LDHs, and their substantial bile acid sequestering ability in the intestinal region. j) Changes in the weight of mice after 4 weeks of treatment with various tablet formulations of LDHs. k) Change in the cholesterol levels in mice after 4 weeks of treatment with various tablet formulations. Reprinted with the permission from Ref.[113] Copyright 2020, Elsevier. l) Blood glucose values of normoglycemic rats (N), diabetic control (DC), diabetic orally treated with GB (5 mg kg^−1^), NP LDH+GB, and LDH after 28 days of treatment. Reprinted with the permission from Ref.[114] Copyright 2023, Elsevier.

Effective drug delivery to the posterior segment of the eye and maintaining therapeutic levels via ocular topical administration remains a significant challenge. A recent study designed a nanocomposite based on carboxymethyl chitosan (CMCS) and LDH to enhance the cellular uptake of nanocomposite eye drops.^[^ ^104^ ^]^ The researchers modified and functionalized CMCS into CMCS‐glutathione‐glycylsarcosine (CMCG‐GS) to create the hybrid nanocomposite ocular drug delivery system, and dexamethasone sodium phosphate (DEXP) was chosen as the drug candidate to study the feasibility of the system (**Figure** **4**a). Compared to commercial products, the bioavailability of CMCG‐GS‐DEXP‐LDH (10:1) nanocomposite eye drops was significantly improved, and DEXP was effectively delivered to the retina through the conjunctival‐scleral pathway (Figure 4b) and remained at effective therapeutic concentrations for a longer time. It is worth noting that hydroxymethyl biopolymer is highly soluble in water and has abundant carboxyl and hydroxyl functional groups, commonly used to prepare pH‐responsive hydrogel‐based drug carriers. In another study, pH‐responsive carboxymethylcellulose (CMC)/LDHs hydrogel was prepared for the delivery of the oral drug amoxicillin.^[^ ^105^ ^]^ LDH played a crucial role in cross‐linking the hydrogel network, thereby increasing the drug loading and regulating drug release by increasing the porosity of the hydrogel network. CMC/LDH(Cu/Al 7.5) bionanocomposite hydrogel beads showed higher swelling and better drug release ability (Figure 4c,d). In this study, LDH served as a modifier of the CMC hydrogel, while drug molecules were carried in the hydrogel spatial network formed by CMC and LDH, released based on the pH responsiveness of the hydrogel system. Although CMC/LDH(Cu/Al 7.5) achieved a good drug loading amount (73%), the researchers did not fully utilize the drug loading performance of LDH itself.

Drug delivery through the oral route is an attractive option due to its convenience, minimal pain, and suitability for chronic therapy.^[^ ^106^ ^]^ However, traditional oral drug delivery systems, such as capsules, typically exhibit low bioavailability.^[^ ^107^ ^]^ Furthermore, long‐term repeated administration may impose a burden on the stomach and even cause side effects.^[^ ^108^ ^]^ To address these issues, it is necessary to develop effective and safe oral drug carriers. Among them, layered double hydroxides (LDHs) stand out for their biological safety, oral tolerability, and fewer adverse reactions compared to other inorganic nanoparticles.^[^ ^109^ ^]^ In addition, LDHs possess excellent properties such as high loading capacity, pH‐responsiveness, and controlled release, making them ideal carriers for oral drugs. Nevertheless, LDHs may dissolve under gastric acid conditions,^[^ ^110^ ^]^ and smaller‐sized nanoscale LDHs may exhibit the issue of drug burst release.^[^ ^99^ ^]^ Therefore, appropriate strategies are required to modify and design LDHs. Recently, some studies have beneficially explored LDHs as an oral drug delivery system. In a recent study, the non‐steroidal anti‐inflammatory drug, diclofenac sodium (Dic), was intercalated into the interlayer of MgAl LDH, and the resulting drug‐loaded LDH‐Dic was encapsulated with alginate (Alg) gel to form Alg/LDH‐Dic beads for oral delivery of Dic.^[^ ^111^ ^]^ As shown in Figure 4e, Alg/LDH‐Dic beads exhibited pH‐responsive release characteristics, and the amount of release of Dic increased with increasing pH of the release matrix (from 29% at pH 2.5 to 64% at pH 12). The swelling of Alg gel with increasing pH was the main reason for the pH‐responsive release behavior of Alg/LDH‐Dic beads. Interestingly, another study fabricated Alg beads implemented with CuAl LDH‐supported copper metal‐organic framework beads (Alg‐DOX‐Cu MOF‐LDH beads) for oral delivery of the anticancer drug doxorubicin (DOX) (Figure 4f).^[^ ^112^ ^]^ Compared to the bare drug carrier (DOX‐Cu MOF‐LDH), the Alg‐encapsulated drug carrier (Alg‐DOX‐Cu MOF‐LDH) exhibited a lower release rate of DOX, achieving a controlled release effect for DOX under the same release conditions (Figure 4g). Moreover, Alg encapsulation diminished the undesired burst release problem of the bare drug carrier in a neutral environment. Additionally, the proposed drug delivery system achieved higher drug release under mildly acidic conditions, which is beneficial for targeted delivery of anticancer drugs. A comparison of Figure 4e and Figure 4g revealed that the two drug‐loading systems based on LDH and Alg constructed by these two studies exhibited completely different pH‐responsive release characteristics. Specifically, Alg/LDH‐Dic beads achieved higher drug release under more alkaline conditions, while Alg‐DOX‐Cu MOF‐LDH beads obtained higher drug release under more acidic conditions. This was because the swelling rate of Alg‐DOX‐Cu MOF‐LDH was completely opposite to that of Alg/LDH‐Dic, with the swelling rate of Alg‐DOX‐Cu MOF‐LDH being higher under acidic than alkaline conditions. The differences in the type, size, and amount of LDH used in the two studies indicated that the addition of LDH significantly affects the swelling behavior of the Alg gel systems and hence its drug release. Further studies are needed to explore the mechanisms involved, but the tunable and controllable drug release of LDH/Alg‐based drug delivery systems by regulating LDH represents an interesting and attractive research focus.

In addition, there were two studies that used enteric co‐polymer, Eudragit S‐100, to wrap LDH tablets for the oral therapy of hyperlipidemia and diabetes, respectively. The Eudragit S‐100 coating prevented the degradation of LDH drug carriers in the stomach and ensured their release in the intestine, while also protecting the intestinal wall from the toxic effects of degraded metal oxides. In Figure 4i, Lin et al.^[^ ^113^ ^]^ prepared surface‐functionalized LDH as an intestinal bile acid sequestrant to reduce hyperlipemia. Notably, LDH was not used as a drug carrier in this study, but rather as a cationic nanocontainer to load bile acids based on its anion exchangeability and high loading capacity. Furthermore, conducting silane modification of LDHs before applying enteric coating improved their stability under acidic conditions. Therefore, the treatment effect of LDHs‐G0‐S100 (LDHs‐N3‐S100) was superior to that of LDHs‐S100 (Figure 4j,k). In another study,^[^ ^114^ ^]^ glibenclamide (GB) LDH drug carriers encapsulated in Eudragit S‐100 (NP LDH+GB) achieved remarkable therapeutic effects in lowering blood glucose values in diabetic rats (Figure 4l).

#### 2.1.3 Non‐Anionic Drug Delivery

Although LDHs have been extensively studied as drug delivery systems for some time, most of these systems have carried anionic drug molecules due to the limitations of the LDHs structure. The positively charged laminates of LDHs, composed of metal cations, require negatively charged anions between the layers to maintain structural stability, and the LDHs drug‐loading system is primarily based on anion exchange to intercalate the target drug molecules into its interlayer. However, current research on the application of LDHs to non‐anionic drug delivery systems is very limited, greatly restricting the potential applications of LDHs as drug delivery systems. We summarize representative works on LDHs used in non‐anionic drug delivery systems to date in **Table** **1**.

**Table 1:** LDHs for hydrophobic drug delivery applications.

Material	Drug	Auxiliary medium	Loading Method	Drug location	Loading rate [%]	Reference
MgAl NO_3_ LDH	Camptothecin	Sucrose aspartate	Ion exchange	Interlayer	5.6	[115]
MgAl NO_3_ LDH	Camptothecin	Sodium cholate	Ion exchange	Interlayer	0.9	[115]
MgAl NO_3_ LDH	Gramicidin	Sodium cholate	Ion exchange	Interlayer	2.2	[116]
MgAl NO_3_ LDH	Ellagic acid		Coprecipitation	Interlayer	50	[117]
MgAl NO_3_ LDH	Doxorubicin		Adsorption	Surface	26.4	[78]
MgAl NO_3_ LDH	Doxorubicin	Poly(acrylic acid) sodium salt	Ion exchange	Interlayer	32.0	[118]

**Figure 5:**
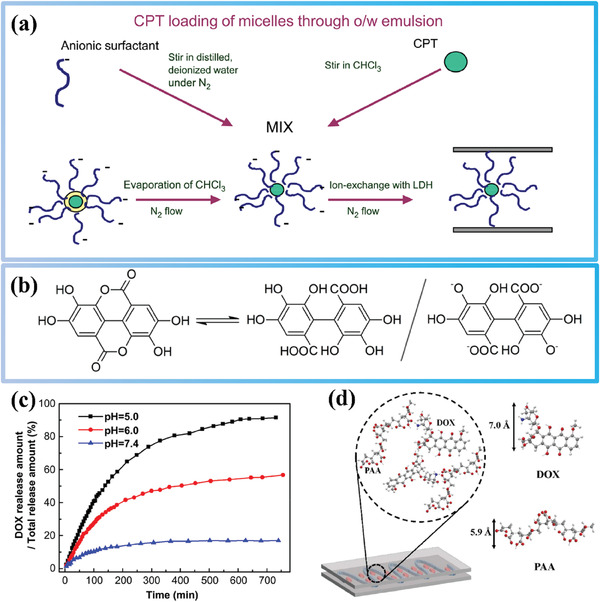
a) Schematic preparation process of nanobiohybrids as delivery vehicles for camptothecin (CPT). Reprinted with the permission from Ref.[115] Copyright 2004, Elsevier. b) Chemical equilibrium between EA and its opened form, 4,4′,5,5′,6,6′‐hexahydroxydiphenic acid, and its potential intercalated form. Reprinted with the permission from Ref.[117] Copyright 2020, MDPI. c) In vitro release profiles of LDH‐Gd/Au‐DOX under different pH values. Reprinted with the permission from Ref.[78] Copyright 2013, Elsevier. d) Schematic of the structure of DOX‐PAA‐LDH hybrids. Reprinted with the permission from Ref.[118] Copyright 2021, Elsevier.

To address the challenge of intercalating non‐anionic drugs into the interlayers of LDHs, researchers have proposed using an intermediate drug storage medium. One such medium is a micelle system composed of anionic surfactants, as demonstrated in a 2004 study.^[^ ^115^ ^]^ The micelle system offers an intermediate storage medium for non‐anionic drugs, as the hydrophobic core of the micelle can house the drug while its negatively charged shell can complete anion exchange with LDH, enabling intercalation of the drug into the interlayer of LDH (**Figure** **5**a). In this study, sodium cholate (SC), a common surfactant, and self‐synthesized surfactant sucrose aspartate (SAS) were used at concentrations exceeding their respective CMC concentrations to form micelles in the solution and load the anionic drug camptothecin (CPT). The drug loading rates of LDH‐SAS‐CPT and LDH‐SC‐CPT were 5.6% and 0.9%, respectively, as shown in Table 1. However, the article did not analyze the reason for the difference in the drug loading rate of the two LDH‐based micellar systems. We speculate that this may be due to the fact that the micelles formed by SAS are smaller than those formed by SC, resulting in more drug‐loaded SAS micelles that can be intercalated between LDH layers. Nevertheless, limited information is available on SAS surfactant, and despite providing details on the synthesis method of SAS surfactant,^[^ ^119^ ^]^ information on the size of SAS micelles remains unknown. Furthermore, although XRD was used to characterize the interlayer spacing difference of LDH before and after loading of the drug‐stored intermediate medium, the study neglected to perform important experimental controls. Since the LDH with only micelles loaded was not characterized, the change in interlayer spacing of LDH does not necessarily indicate that the drug is intercalated into the interlayer of LDH.

In a later study, the non‐anionic drug gramicidin was loaded onto LDH with the help of SC micelles in a similar manner.^[^ ^116^ ^]^ The drug loading rate of LDH‐SC‐gramicidin was higher than that of LDH‐SC‐CPT, which may be due to the higher concentration of SC in the later study, thus forming more micelles for drug loading. The interlayer spacing of LDH‐SC‐gramicidin (3.56 nm) and LDH‐SC (3.39 nm) was characterized by XRD. However, the similarity of the interlayer spacings makes it difficult to prove that the drug molecules are indeed distributed between the LDH layers. Future studies may consider using small‐angle XRD to further characterize the internal spatial structure of the LDH/micelle drug‐loaded system to provide direct evidence of drug intercalation into LDH.

It is worth noting that the drug loading rates of LDH/micelle systems constructed in these studies were not high. The reason is that the diameter of the micellar sphere is relatively large and can occupy a significant amount of space between LDH layers, thereby limiting the drug loading rate of LDH. While micellar drug‐loading intermediary systems offer a possibility for non‐anionic drug intercalation toward LDH, further optimization of experimental conditions and characterization techniques is required in future work.

In a recent study, researchers successfully achieved one‐step loading of the non‐anionic oxidant ellagic acid (EA) by introducing it during the process of preparing LDH through co‐precipitation.^[^ ^117^ ^]^ The LDH‐based hybrids prepared in this study exhibit good antioxidant activity and a high loading rate of EA. However, it is important to note, as depicted in Figure 5b, that structural analysis revealed that the structure of EA transformed into an anionic form of 4,4′,5,5′,6,6′‐hexahydroxydiphenic acid during the intercalation process. Since the layers of LDHs require interlayer anions to maintain balance, the co‐precipitation method used to intercalate non‐anionic drug molecules may lead to changes in their structures, which could potentially affect their functions. Therefore, this study is a valuable reference, but it is crucial to pay close attention to the changes in the drug structures before and after intercalation into LDHs.

Additionally, Several studies have attempted to load the non‐anionic anticancer drug doxorubicin (DOX) onto LDHs.^[^ ^78^, ^118^, ^120^, ^121^, ^122^, ^123^ ^]^ Among them, Wang et al. (2013) first reported the use of layered double hydroxides (LDHs) to load DOX, achieving a loading rate of 26.4%.^[^ ^78^ ^]^ The pH‐responsive release of DOX from the LDH‐based carrier was also demonstrated (Figure 5c), making it advantageous for targeted drug delivery. However, analyzing the results of this study, we may find that the loading of DOX by LDH in this study is based on the adsorption induced by the hydrogen bonding formatted between them. And, the pH‐responsive release of DOX is not mediated by the pH‐responsive property of LDHs itself like other LDHs‐based drug delivery systems. Instead, the pH‐responsive release of DOX in this research is due to the pH‐dependent solubility of DOX itself and its protonation in the acidic environment.^[^ ^124^ ^]^ Notably, prior to this study, only a limited range of anionic anticancer drugs had been loaded onto LDHs. The success of loading DOX onto LDHs in this study has led to many subsequent works using the same approach.^[^ ^120^, ^121^, ^122^, ^123^ ^]^ However, this surface‐loading method cannot accommodate non‐anionic drugs into the interlayer of LDHs, which limits the utilization of the high loading capacity of the interlayer.

Actually, DOX is different from the other three non‐anionic drugs mentioned in this section because the amino group of DOX can be protonated at pH 7 to convert it into a cationic drug.^[^ ^125^ ^]^ Therefore, in fact, the conditions can be controlled to realize the conversion of DOX between non‐ionic drugs and cationic drugs. This property inspired a recent study in which DOX was successfully intercalated into the interlayer of LDH with the help of anionic polymer polyacrylic acid (PAA).^[^ ^118^ ^]^ The researchers first constructed a PAA‐intercalated LDH‐PAA system, where anionic polymers generated additional negative charges after being intercalated between LDH layers. As shown in Figure 5d, cationic DOX was then absorbed into the carboxylate moiety of PAA between LDH layers through charge adsorption. The LDH‐PAA‐DOX drug‐loading system achieved a loading rate of 31.95% for DOX, which is higher than the surface‐loading systems mentioned earlier. This work presents a new approach for intercalating cationic drugs into the interlayer of LDHs. Combining this method with the surface‐loading method may further enhance the loading capacity of LDHs for cationic drugs.

#### 2.1.4 Magnetic Core–Shell Hybrids

In recent years, there has been growing interest in core–shell structures composed of iron oxides and LDHs due to the potential for improved targeted drug delivery resulting from the magnetic properties of iron oxides. However, the relatively low stability of Fe_3_O_4_ magnetic particles has presented a significant challenge. To address this issue, the construction of core–shell structures has been proposed as a promising approach to enhance stability.^[^ ^126^ ^]^ Additionally, core–shell structures can increase the stability of drugs and enable controlled drug release.^[^ ^127^ ^]^ Notably, while the construction of Fe_3_O_4_/LDHs‐based core–shell structures is based on the magnetism of Fe_3_O_4_, there are variations in the design and construction sequence of core–shell structures across different studies.

**Figure 6:**
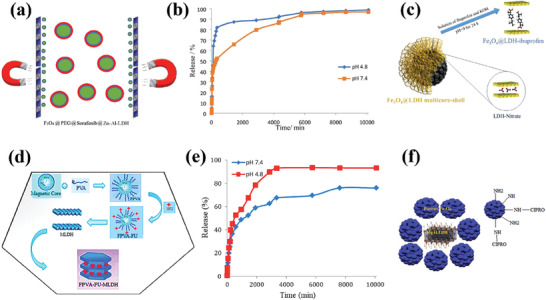
a) Schematic the design of Fe_3_O_4_@PEG@Sorafenib@Zn‐Al‐LDH. Reprinted with the permission from Ref.[128] Copyright 2020, MDPI. b) The sorafenib release profiles from MPVASO‐ZLDH in phosphate‐buffered solutions at pH 4.8 and 7.4. Reprinted with the permission from Ref.[129] Copyright 2021, Elsevier. c) Schematic representation of the preparation of Fe_3_O_4_@LDH multicore–shell nanostructure before and after the intercalation of ibuprofen. Reprinted with the permission from Ref.[130] Copyright 2020, Springer. d) Schematic representation of the core–shell structure of magnetic FPVA‐FU‐MLDH. e) The cumulative release profiles of 5‐fluorouracil from its FPVA‐FU‐MLDH nanoparticles in phosphate‐buffered solution at pH 4.8 and pH 7.4. Reprinted with the permission from Ref.[131] Copyright 2019, MDPI. f) Schematic the design of MgAl‐LDH@PMN‐NH_2_. Reprinted with the permission from Ref.[132] Copyright 2020, Elsevier.

In a recent study, Fe_3_O_4_ was used as the core, and its surface was modified with polyethylene glycol (PEG) to enhance the stability of the magnetic core.^[^ ^128^ ^]^ Drug anions were then adsorbed on Fe_3_O_4_‐PEG via low electrostatic attraction, followed by further coating with LDH (**Figure** **6**a). Another study employed a similar approach to design Fe_3_O_4_/LDH‐based core–shell hybrids but instead used polyvinyl alcohol (PVA) to modify the surface of Fe_3_O_4_.^[^ ^129^ ^]^ In both studies, LDHs served as a coating to improve the biocompatibility of the fabricated core–shell hybrids and delay the release of the drug encapsulated inside, thereby achieving controlled drug release. The LDHs acted as a physical protection barrier in these two studies. However, the controlled release effect of the drug was limited, and burst release was observed during the early stage of drug release (Figure 6b). Furthermore, the pH‐responsive properties of the drug were poor, as the pH‐responsive release of the drug was determined by the drug itself rather than the drug carrier.

Additionally, a core–shell hybrid composed of Fe_3_O_4_ and LDHs was designed in another study, where Fe_3_O_4_ was used as the core and LDH as the shell.^[^ ^130^ ^]^ However, the LDH shell in this study was drug‐loaded, with drug molecules intercalated between the LDH layers (Figure 6c). Since the focus of this study was on the delivery of anti‐inflammatory drugs, the release of drug carriers was only studied at pH 7.4. Due to the smaller size of the LDH used (70–110 nm) and the burst release of smaller LDH drug‐loaded particles, the controlled release behavior of the drug carrier in this study was not optimal. Interestingly, another study also used Fe_3_O_4_ as the core, but the drug‐loaded magnetic core was intercalated into the interlayer of the LDH as a whole^[^ ^131^ ^]^ (Figure 6d). Additionally, due to the pH‐responsive release characteristics of LDH itself, the pH‐responsive release behavior of the core–shell hybrid constructed in this study was more prominent (Figure 6e). However, due to the small size of the core–shell system (31 nm), the drug carrier still exhibited burst release behavior in the early stage of release. Notably, another Fe_3_O_4_/LDH‐based core–shell system was constructed in a separate study, where LDH was used as the core and Fe_3_O_4_ as the shell (Figure 6f).^[^ ^132^ ^]^ Furthermore, Fe_3_O_4_ was modified using a silane coupling agent, and the drug molecules were connected to the surface of the core–shell structure through hydrogen bonding. Therefore, the release of drugs in the drug carrier in this study depended on the strength of the hydrogen bond between the drug itself and the carrier, which was affected by the release environment.

In general, research on the fabrication of Fe_3_O_4_/LDHs‐based core–shell hybrids provides a new direction and innovative ideas for the construction of efficient and controlled‐release drug carriers. However, there is still significant room for improvement in related works. For instance, these core–shell drug delivery systems typically exhibit burst drug release behaviors, and there is a lack of research evaluating the stability of the core–shell systems over time. It is noteworthy that related works have successfully constructed Fe_3_O_4_/LDHs‐based core–shell hybrids and confirmed their good biocompatibility. However, no research has realized the use of an external magnetic field to control the magnetic core–shell drug carrier for in vivo studies, which may be because of difficulties in precisely controlling the magnetic hybrids. Therefore, future research on LDH‐based magnetic core–shell hybrids should aim for higher requirements, such as preparing biosafe drug carriers with high drug loading and high dimensional stability that can be precisely controlled using an external magnetic field.

### 2.2 LDHs for Cancer Diagnosis and Therapy

LDHs have long been of great interest in cancer‐related research. LDHs can accumulate in tumor tissues through the enhanced permeability and retention (EPR) effect,^[^ ^133^ ^]^ and the micro‐acidity of the tumor microenvironment (TME) can promote the rapid release of drugs or functional molecules from the LDH carrier.^[^ ^134^ ^]^ This greatly enhances the biological utilization and targeting of drug molecules, and effectively reduces the toxic side effects of anticancer drugs on normal human tissues, providing a safer and more effective method for cancer treatment. In recent studies on the use of LDHs in cancer treatment, drugs such as doxorubicin (DOX),^[^ ^120^, ^121^, ^122^, ^135^ ^]^ 5‐fluorouracil (5FU),^[^ ^136^, ^137^ ^]^ paclitaxel (PTX),^[^ ^136^ ^]^ Dihydroartemisinin (DHA),^[^ ^138^ ^]^ and methotrexate (MTX)^[^ ^139^ ^]^ are commonly used.

Due to the controllable and modifiable structure of LDHs, they have been not only studied for traditional chemotherapy (CT),^[^ ^121^, ^136^ ^]^ but have also been studied in some emerging cancer therapies, such as immunotherapy,^[^ ^140^, ^141^, ^142^ ^]^ chemodynamic therapy (CDT),^[^ ^138^, ^143^, ^144^, ^145^, ^146^, ^147^, ^148^ ^]^ photodynamic therapy (PDT),^[^ ^120^, ^143^, ^145^, ^147^, ^149^, ^150^ ^]^ photothermal therapy (PTT),^[^ ^122^, ^124^, ^135^, ^137^, ^138^, ^143^, ^144^, ^146^, ^147^, ^148^, ^151^ ^]^ and sonodynamic therapy (SDT).^[^ ^152^, ^153^ ^]^ In addition to the targeted delivery of specific anti‐cancer drugs, LDH nanomedicines that accumulate within tumor tissues have also demonstrated their potential as adjuvants for inducing personalized in situ immune responses, highlighting their applicability in immunotherapy.^[^ ^154^ ^]^ Immunotherapy represents a form of cancer treatment that focuses on modulating the immune system rather than directly targeting the tumor.^[^ ^155^ ^]^ Typically, adjuvants are employed to enhance the immunogenicity of antigens and activate antigen‐presenting cells (APCs).^[^ ^156^ ^]^ Remarkably, LDHs have emerged as favorable adjuvants for immunotherapy, owing to their layered structure, positively charged host layers, and tunable interlayer spacing, enabling them to efficiently load substantial antigen quantities and interact with APCs or lymphoid organs, ultimately promoting robust and enduring immune responses.^[^ ^48^, ^75^, ^154^ ^]^ Zhang et al.^[^ ^142^ ^]^ developed an immunomodulatory adjuvant utilizing Zn‐doped LDH (Zn LDH). Peritumoral injection of Zn LDH results in continuous pH neutralization within the TME and the release of significant amounts of zinc, fostering an inflammatory network comprising cytotoxic T cells, M1 tumor‐associated macrophages, and natural killer cells. Moreover, Zn LDH disrupts endo‐/lysosomes to inhibit autophagy and induce mitochondrial damage. The liberated zinc activates the cGAS‐STING signaling pathway, thereby instigating immunogenic cell death. Another recent advancement in this field involves the development of an LDH‐based enhancer of immunogenic cancer cell death. A construct known as Cu‐LDH, decorated with FeOOH nanodots and loaded with a heat shock protein inhibitor (FeOOH@STA/Cu‐LDH), was engineered and employed as a nanoamplifier for tumor immunogenic cell death.^[^ ^141^ ^]^ In both in vitro and in vivo 4T1 tumor models, FeOOH@STA/Cu‐LDH exhibited efficient photothermal properties at fever‐like temperatures (40–42 °C). This enhanced the generation of reactive oxygen species (ROS) catalyzed by FeOOH nanodots within the TME, thereby significantly promoting the translocation of calreticulins (CRT) to the cell membrane, inducing apoptosis in cancer cells, and stimulating cytotoxic T lymphocytes (CTLs) to initiate a systemic anti‐tumor immunogenic response.

It is known that H_2_O_2_ can be enriched in tumor tissue and cancer cells,^[^ ^157^ ^]^ and the Fenton effect can reduce H_2_O_2_ to generate hydroxyl radicals, which can effectively promote the apoptosis of cancer cells.^[^ ^158^ ^]^ CDT and PDT for cancer are based on the hydroxyl radicals generated by the Fenton effect to kill cancer cells and inhibit tumor growth, but they trigger the Fenton reaction in different ways. CDT does not require external energy but triggers the Fenton reaction by Fenton agents or metal ions.^[^ ^159^ ^]^ In recent studies, the Fenton agents integrated with LDHs for CDT include DHA^[^ ^138^ ^]^ and chlorin e6 (Ce6),^[^ ^148^ ^]^ while the metal ions that constitute the LDHs structure, such as Fe ions,^[^ ^144^, ^145^, ^146^, ^147^ ^]^ Cu ions,^[^ ^143^, ^144^ ^]^ Mn ions,^[^ ^146^, ^147^ ^]^ and Co ions,^[^ ^146^ ^]^ can also trigger the Fenton reaction. PDT operates by leveraging photosensitizers to induce the generation of highly cytotoxic ROS through two distinct mechanisms.^[^ ^160^ ^]^ These two PDT mechanisms diverge in their pathways involving the electronically excited state, predominantly the long‐lived lowest triplet excited state (T_1_), of the photosensitizers as they interact with O_2_. Within the Type I pathway, photosensitizers facilitate the transfer of electrons to O_2_, resulting in the formation of superoxide anion radicals (O_2_^−^). Conversely, in the Type II pathway, photosensitizers channel energy toward O_2_, leading to the creation of singlet oxygen molecules (^1^O_2_). Typically, the I‐type and II‐type processes occur simultaneously during PDT.^[^ ^161^ ^]^ Notably, the practical application of PDT encounters impediments originating from the restricted tissue penetration capabilities of visible light (1–6 mm) and the relatively inefficient generation of ^1^O_2_ by photosensitizers.^[^ ^162^ ^]^ The limited penetration depth of visible light arises from its absorption by intrinsic chromophores such as hemoglobin and cytochromes within biological tissues, alongside phenomena like light scattering, diffusion, and disorientation, which are consequences of the complex and heterogeneous structure of biological tissues.^[^ ^163^ ^]^ In light of these impediments, the near‐infrared (NIR) light spectrum, encompassing NIR‐I (700–1000 nm), NIR‐II (1000–1350 nm), and NIR‐III (1350–1870 nm), emerges as the preferred choice due to its designation as the “optical window” within biological tissue, which significantly mitigates tissue scattering.^[^ ^164^ ^]^ Consequently, NIR light stands as the prevalent choice for serving as the light source in the context of LDHs employed for PDT applications.^[^ ^120^, ^143^, ^145^, ^147^, ^150^ ^]^ And, indocyanine green (ICG)^[^ ^120^, ^143^, ^145^ ^]^ and Ce6^[^ ^147^, ^150^ ^]^ are frequently utilized as photosensitizers when combined with layered double hydroxides (LDHs). Furthermore, specific compositions of LDHs, such as CoMo‐LDH, can also be employed as photosensitizers through etching.^[^ ^149^ ^]^

PTT is a method that uses the photothermal effect to kill tumor cells.^[^ ^165^ ^]^ When the photothermal agent is excited, the absorbed energy is converted into heat, leading to local heating. Tumor cells have a low tolerance for temperature, and when the local temperature rises to a certain degree, the tumor cells are killed.^[^ ^166^ ^]^ Recently, photosensitizers such as ICG^[^ ^120^, ^143^, ^148^ ^]^ and gold nanoparticles (AuNP)^[^ ^135^ ^]^ have been used in combination with LDHs. In addition, LDHs have a layered structure and a controllable chemical composition, and the metal ions in them can also be excited to produce a photothermal effect.^[^ ^167^, ^168^ ^]^ Common metal ions that can be used for this purpose are Co,^[^ ^146^, ^151^ ^]^ Fe,^[^ ^122^, ^138^, ^144^, ^146^, ^147^, ^151^ ^]^ Mn,^[^ ^138^, ^146^, ^147^ ^]^ and Cu^[^ ^120^, ^137^, ^144^ ^]^ ions. It should be noted that the selection and control of metal ions is a key issue in photothermal therapy research, and it needs to be based on specific treatment needs and material characteristics. In addition, in the application of LDHs to PTT and PDT, defect engineering has attracted the attention of researchers.^[^ ^143^, ^149^, ^151^ ^]^ The defect engineering involves chemically etching the surface of LDHs to form micro‐ and nanostructures of certain depths and shapes, thereby achieving control over the optical properties such as light absorption, scattering, and local field intensity of LDHs, which can enhance the effectiveness of LDHs in PDT and PTT.^[^ ^169^ ^]^ It is worth noting that etched LDHs have recently been discovered to hold potential applications in SDT. SDT is an emerging noninvasive therapeutic modality designed to induce the production of ROS.^[^ ^170^ ^]^ SDT leverages the use of exogenous ultrasound to activate sonosensitizers, which exhibit notable features such as precise targeting and minimal impact on normal tissue.^[^ ^171^ ^]^ Compared to PDT and PTT, ultrasound‐mediated SDT offers superior tissue penetration capabilities, making it advantageous for controlled non‐invasive treatment of deep‐seated or large tumors.^[^ ^172^ ^]^ Recently, Hu et al.^[^ ^152^ ^]^ achieved a phase transition in CoW LDH and NiW LDH nanosheets, shifting them from a polycrystalline to an amorphous state through a straightforward acid etching procedure. These amorphous nanosheets were employed as highly efficient sonosensitizers for SDT. The phase transformation and alterations in the electronic structure induced defect formation, resulting in enhanced ROS generation capabilities in the amorphous CoW LDH nanosheets when exposed to ultrasound irradiation, consequently leading to effective tumor eradication.

In addition, LDHs can also be used as diagnostic systems for cancer imaging. The layer structure of LDHs can be tuned by changing parameters such as composition, size, and morphology to achieve tunable physical and chemical properties to meet the needs of different imaging modes. Recent work on the application of LDHs in tumor imaging mainly involves magnetic resonance imaging (MRI),^[^ ^122^, ^123^, ^147^, ^148^, ^150^, ^173^ ^]^ computed tomography imaging (CTI),^[^ ^139^, ^147^ ^]^ fluorescence imaging,^[^ ^147^ ^]^ and photoacoustic imaging (PAI).^[^ ^148^, ^150^ ^]^ Metal ions (such as Cu^2+^,^[^ ^148^, ^173^ ^]^ Fe^2+^,^[^ ^122^ ^]^ Fe^3+^,^[^ ^147^ ^]^ Mn^2+^,^[^ ^147^, ^150^ ^]^ Co^2+^,^[^ ^150^ ^]^ etc.) or doped rare earth elements (such as Gd,^[^ ^123^, ^173^ ^]^ Dy,^[^ ^123^ ^]^ etc.) in LDHs have unpaired electrons, which will affect the surrounding magnetic field, thereby changing the magnetic field strength in that area. When LDHs are used as MRI contrast agents, this change can be detected and converted into an increase in the intensity of the MRI signal. By doping radioactive isotopes (such as Co‐57^[^ ^139^ ^]^) or rare earth elements (such as Yb^[^ ^147^ ^]^) into the structure of LDHs, they can have excellent X‐ray absorption and scattering ability and can be used as CTI contrast agents. The high surface area of LDHs also provides a large number of binding sites for fluorescent dyes (such as chlorin e6 (Ce6)^[^ ^147^ ^]^), making them useful as fluorescent imaging probes. Additionally, based on the optical and acoustic properties of LDHs, they can be used for emerging PAI. Specifically, when LDHs are irradiated by a laser beam, they generate heat and cause instantaneous expansion and contraction in the local area, resulting in photoacoustic signals. These photoacoustic signals can be captured by an ultrasound detector and converted into images, thus achieving imaging of the area where LDHs are located.^[^ ^150^ ^]^

Enhancing the accumulation of LDHs at the site of lesions is a crucial goal in driving their clinical applications, which directly impacts the efficiency of treatments and diagnostics of LDHs while reducing their systemic toxicity.^[^ ^174^ ^]^ To achieve this objective, various strategies have been employed, but there are still several challenges that exist. First, through the EPR effect strategy, researchers utilize the unique vascular structure within tumor tissues, characterized by irregularly arranged defective endothelial cells and larger endothelial pores, to enable passive targeting and accumulation of LDHs of ≈100 nm in size.^[^ ^175^, ^176^ ^]^ This strategy allows LDHs to selectively penetrate and reside within tumor tissues, thus enhancing the efficiency of treatment and diagnosis. However, this strategy still has limitations in some cases, especially due to variations in tumor angiogenesis among different tumor types and the presence of high interstitial fluid pressure in solid tumors.^[^ ^177^ ^]^ To overcome these limitations, the development of active targeting strategies for LDHs becomes crucial. By modifying the surface of LDHs with ligands possessing specific targeting properties, such as folic acid (FA), interactions can be established with folate receptors overexpressed on tumor cells, thus augmenting the accumulation of LDHs at the tumor site and further improving therapeutic effect.^[^ ^178^ ^]^ Nevertheless, even with these strategies in place, there are still several challenges to be addressed. First, different tumor types exhibit heterogeneous biological characteristics, rendering a single strategy potentially ineffective for all cases and necessitating personalized research and treatment approaches. Additionally, biological barriers, including peritumoral and intratumoral blood vessels, extracellular matrix, and the blood‐brain barrier, may limit the penetration and accumulation of LDHs, requiring further technological advancements to overcome these barriers.^[^ ^179^, ^180^ ^]^ Lastly, ensuring the biocompatibility and toxicity profile of LDHs and their modifications is a crucial consideration, necessitating extensive research and safety assessments. In summary, enhancing the accumulation of LDHs at lesion sites is pivotal for improving their efficiency in treatments and diagnostics, but it requires overcoming various biological barriers and limitations for better clinical applications. By comprehensively applying passive targeting, active targeting, and strategies to overcome biological barriers, the accumulation of LDHs at lesion sites can be maximized, thereby enhancing their potential for clinical applications.

Additionally, it is worth noting that by modifying and functionalizing the surface of LDHs, their targeting ability, drug release performance, stability, and biocompatibility in tumor cells can be improved.^[^ ^181^ ^]^ Therefore, surface modification of LDHs is particularly necessary before using them for cancer therapy. Surface modification can increase the affinity and specificity of LDHs on the surface of tumor cells, thereby improving drug targeting and selectivity.^[^ ^182^ ^]^ The common strategy for targeting‐specific modification of LDHs is to modify their surface with FA, which can increase LDHs’ recognition and binding ability to cancer cells, thereby increasing the local concentration of drugs in the tumor, and reducing side effects, and toxicity.^[^ ^123^ ^]^ In addition, surface modification can affect the drug release performance of LDHs, such as regulating the release rate of drugs, thereby achieving better therapeutic effects.^[^ ^121^ ^]^ More importantly, surface modification can improve the biocompatibility and stability of LDHs, reducing their risk of immune rejection and side effects in the biological systems.^[^ ^183^ ^]^ Currently, commonly used approaches for surface wrapping of LDHs include polyaniline (PANI),^[^ ^121^ ^]^ PEG,^[^ ^144^, ^145^, ^147^, ^149^ ^]^ and BSA,^[^ ^120^, ^136^, ^137^, ^143^ ^]^ or combing LDHs with biocompatible chitosan^[^ ^135^ ^]^ and gelatin hydrogel^[^ ^146^ ^]^ to enhance their biocompatibility and stability, reducing the specific binding of LDHs with proteins and cells in the biological systems, thereby reducing their risk of immune rejection and side effects.

In summary, LDHs have outstanding potential for applications in cancer treatment and diagnosis, and representative recent works are summarized in **Table** **2**. In the following sections, we will review the progress and breakthroughs of LDHs in the fields of cancer treatment and diagnosis from three aspects: monotherapy, synergistical therapy, and nanotheranostics, based on recent research.

**Table 2:** Recent work about related LDHs for diagnosis and therapy.

LDHs	Drug	Functionalized molecule	Cancer Type	Functions	Reference
MgAl CO_3_ LDH	DOX	Mn_3_O_4_; N‐graphene quantum dots; Polyaniline	MCF7 Cells	CT	[121]
MgAl NO_3_ LDH	5FU and PTX	Albumin	HCT‐116 cells	CT	[136]
CoMo NO_3_ LDH		PEG	4T1 cells	PTT	[149]
MgAlZn LDH			B16F10 and 4T1 cells	Immunotherapy	[142]
CoW LDH/NiW LDH			4T1 cells	SDT	[152]
CoFe NO_3_ LDH			HeLa cells	PTT/MRI imaging/PA imaging	[151]
MnMgFe Cl LDH	DHA		4T1 cells	PTT/CDT	[138]
MgAl NO_3_ LDH	DOX	3‐isocyanatopropyltriethoxysilane; Folic acid conjugated thiolated chitosan; AuNP	MCF7 Cells	CT/PDT	[135]
CuMgAl Cl LDH		ICG	4T1 cells	PTT/PDT/CDT	[143]
CuMgAl Cl LDH	DOX	ICG and BSA	B16F0 cells	CT/PTT/PDT	[120]
CuMgAl Cl LDH	5‐FU and PTX	BSA	4T1 cells	CT/PDT	[137]
CuFe NO_3_ LDH		Thioacetamide and PEG	HepG2 cells	CDT/PTT	[144]
FeAl Cl LDH		ICG and PEG	4T1 cells	CDT/PDT	[145]
CoMnFe LDO		Glucose Oxidase and Gelatin Hydrogel	4T1 cells	CDT/PTT/Starvation Therapy	[146]
GdCuMgAl Cl LDH			B16F0 cells	MRI	[173]
Cu LDH		FeOOH; Hsp90 inhibitor; BSA	4T1 cells	Immunotherapy/CDT/PTT	[141]
MgAl NO_3_ LDH	MTX	Co‐57	CT‐26 cells	Nanotheranostics Platform based on CTI/CT	[139]
CoMn NO_3_ LDH		Ce6	U87mg, HepG2, 4T1 and Hela cells	Nanotheranostics Platform based on PAI/MRI/Fluorescence Imaging/CDT/PDT	[150]
MnFe Cl LDH		Mesoporous silica and Ce6‐covalently coated upconversion nanoparticles; PEG	HeLa cells	Nanotheranostics Platform based on MRI/CTI/Fluorescence Imaging/PTT/PDT	[147]
Cu/Al NO_3_ LDH		ICG and Ce6	HepG2 cells	Nanotheranostics Platform based on PAI/MRI/CDT/PTT	[148]
Fe/Mg/Al Cl LDH	DOX		4T1 cells	Nanotheranostics Platform based on MRI/CTI/PTT	[122]

#### 2.2.1 Monotherapy

**Figure 7:**
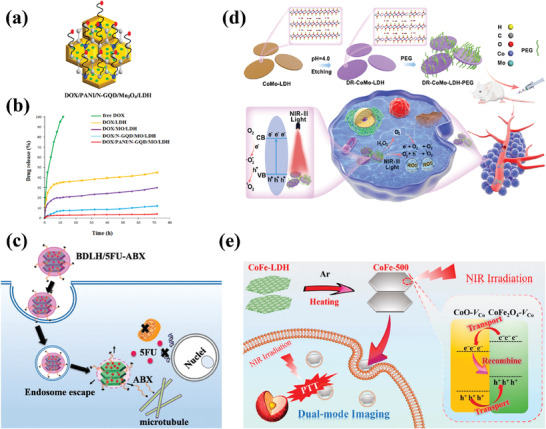
a) Schematic design of DOX/PANI/N‐GQD/Mn_3_O_4_/LDH. b) DOX release from the samples under different simulated physiological conditions of pH 7.4. Reprinted with the permission from Ref.[121] Copyright 2020, Elsevier. c) Schematic illustration of preparation and co‐delivery of 5FU‐ABX‐loaded albumin‐stabilized LDH nanoparticles (BLDH/5FU‐ABX) for colorectal cancer treatment. Reprinted with the permission from Ref.[136] Copyright 2021, Elsevier. d) Schematic illustration of the defect engineering of CoMo‐LDH nanosheets, surface modification with PEG, and its application in NIR‐III PDT. Reprinted with the permission from Ref.[149] Copyright 2020, American Chemical Society. e) Schematic Illustration of the Synthesis of CoFe‐500 from CoFe‐LDH, along with the Mode of Action of CoFe‐500 for NIR Light‐Driven PTT Guided by PA/MR/NIR Imaging. Reprinted with the permission from Ref.[151] Copyright 2022, Nature.

CT remains the most widely used cancer treatment strategy.^[^ ^184^ ^]^ As mentioned in Section 2.1.3, current loading strategies for non‐anionic anticancer drug DOX on LDHs often involve surface loading, which leads to issues such as low drug encapsulation efficiency and burst release. In a recent study,^[^ ^121^ ^]^ researchers co‐modified the surface of LDHs with N‐graphene quantum dots (N‐GQD), Mn_3_O_4_, and polyaniline (PANI) (**Figure** **7**a). The modified LDH showed significantly improved biocompatibility, drug loading capacity, and drug encapsulation efficiency. Moreover, under normal physiological conditions (pH 7.4), the interaction between DOX and the modified LDH was strengthened, resulting in prolonged retention of most DOX in the PANI/N‐GQD/MO/LDH nanocomposite. As shown in Figure 7b, at pH 7.4, the release rate of DOX from the PANI/N‐GQD/MO/LDH was very slow, with a release rate of only ≈4% after 72 h. However, in a mildly acidic environment, DOX can be rapidly released based on its own protonation and pH‐induced solubility, which is beneficial for effective cancer treatment. In another study,^[^ ^136^ ^]^ researchers fabricated an albumin‐stabilized LDH drug carrier for the dual delivery of two anticancer drugs, 5FU and human serum albumin‐bound PTX (ABX) (Figure 7c). Due to the different mechanisms of action of 5FU and ABX in cancer cell inhibition, the use of an LDH carrier for the co‐delivery of the two drugs can achieve good synergistic therapeutic effects, thereby improving the efficiency of cancer treatment. The result showed that the BLDH/5FU‐ABX nanodrug constructed in this study synergistically inhibited HTC‐116 tumor cell proliferation and induced cell apoptosis by increasing intracellular ROS levels and cell cycle arrest. In addition, other works were carried out to transform CoFe‐LDH and CoMo‐LDH into photo‐triggers suitable for PTT and PDT, respectively, through defect engineering strategies. Under the trigger of NIR light at specific wavelengths, CoMo‐LDH^[^ ^149^ ^]^ and CoFe‐LDH^[^ ^151^ ^]^ can induce photodynamic reactions and photothermal effects, respectively, resulting in the apoptosis of cancer cells (Figure 7d,e). It is worth noting that in both studies, LDHs only serve as photo‐reactive triggers without any anti‐cancer drugs loaded, which provides a new, safe, and reliable approach for cancer treatment without drugs.

#### 2.2.2 Synergistical Therapy

As the name suggests, synergistic therapy refers to the combination of multiple cancer treatment modalities to achieve a safer and more effective therapeutic effect. Monotherapy may have deficiencies in efficiency and stability. For example, the low bioavailability of anti‐cancer drugs may increase the risk of CT with higher drug doses, and long‐term CT may lead to significant side effects and drug resistance.^[^ ^185^ ^]^ On the other hand, the efficacy of CDT may be severely limited by the low level of H_2_O_2_ in the TME and its mildly acidic nature.^[^ ^186^ ^]^ While, PDT primarily relies on the production of singlet oxygen (^1^O_2_) in tissue, and the continuous consumption of oxygen can worsen tumor hypoxia and result in inadequate PDT performance.^[^ ^187^ ^]^ In addition, the excessive expression of glutathione (GSH) in the TME tends to eliminate ROS, making cancer cells highly adaptable to oxidative stress and reducing the efficiency of ROS‐mediated cancer cell death, which greatly limits the efficacy of CDT and PDT.^[^ ^188^ ^]^ Moreover, the low penetration depth, inadequate photothermal conversion efficiency, and short circulation time significantly hinder further application of PTT in cancer treatment.^[^ ^189^ ^]^ Therefore, developing new LDH platforms that can combine multiple monotherapies has become a hot topic and focus of current research in order to synergize the effectiveness of monotherapies.

**Figure 8:**
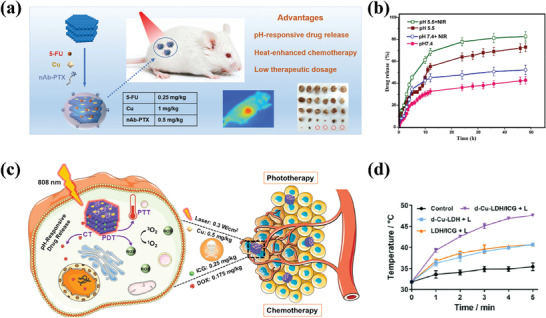
a) Schematic design of 5FU/Cu‐LDH@nAb‐PTX for synergistical chemo‐photo‐therapy. Reprinted with the permission from Ref.[137] Copyright 2021, Elsevier. b) Cumulative DOX release profile at pHs 5.5 and 7.4 in the absence and presence of NIR radiation. Reprinted with the permission from Ref.[135] Copyright 2020, Elsevier. c) Schematic Illustration of Multifunctional ICG/Cu‐LDH@BSA–DOX Nanomedicine. Reprinted with the permission from Ref.[120] Copyright 2021, American Chemical Society. d) Skin temperature profiles of tumor‐bearing mice in different groups under 808 nm laser irradiation (+L) at the power density of 0.23 W cm^−2^ (*n*  =  3). Reprinted with the permission from Ref.[143] Copyright 2020, Wiley.

By combining different treatment methods, it is possible to achieve effective and safe cancer treatment under milder conditions, lower drug concentrations, and even non‐pharmacological approaches. In Section 2.2.1, a study was mentioned that used LDH co‐loaded with 5 FU and PTX to achieve delivery of both drugs.^[^ ^136^ ^]^ The therapeutic concentrations of 5FU and PTX used in this study were 3 and 20 mg kg^−1^, respectively. Building on this work, the team in another study pre‐treated LDH with Cu deposition before loading the two drugs (**Figure** **8**a), as Cu ions can trigger a photothermal effect.^[^ ^137^ ^]^ The constructed 5Fu/Cu‐LDH@nAb‐PTX can accumulate in tumor tissues under the EPR effect, and the acidic environment of the TME promotes rapid drug release. 5Fu/Cu‐LDH@nAb‐PTX also degrades under slightly acidic conditions, releasing copper ions. By applying infrared light (808 nm, 0.75 W cm^−2^, 3 min), the photothermal effect generated by copper ions not only kills cancer cells but also promotes the faster release of the drug carrier. Results from another study have also shown that the drug release behavior of LDH carriers is significantly accelerated under NIR light irradiation^[^ ^135^ ^]^ (Figure 8b). The 5 Fu/Cu‐LDH@nAb‐PTX constructed in this study is an excellent pH‐sensitive agent for drug release, which can effectively synergize PTT and CT. Therefore, by doping only a small amount of copper ions on LDH, the drug concentration required to achieve therapeutic effects can be significantly reduced (0.25 mg kg^−1^ 5 FU, 1 mg kg^−1^ Cu, 0.5 mg kg^−1^ PTX) (Figure 8a). However, it should be noted that the laser fluence (0.75 W cm^−2^) of the laser applied in this study exceeds the safety standard (0.33 W cm^−2^) set by the American National Standards Institute (ANSI),^[^ ^190^ ^]^ which poses certain safety risks. This is because relying solely on the metal ions of the LDHs structure to trigger the PTT may be limited in efficiency. Therefore, in another work, the photosensitizer ICG was integrated into Cu‐LDH.^[^ ^120^ ^]^ It is worth mentioning that both ICG and Cu‐LDH can trigger photodynamic and photothermal effects. Thus, the ICG/Cu‐LDH@BSA‐DOX constructed in this study can achieve synergistic CT, PTT, and PDT (Figure 8c). In vivo experiments have shown that it can almost eliminate tumor tissue with low‐dose treatment (0.175 mg kg^−1^ DOX, 0.5 mg kg^−1^ Cu, 0.25 mg kg^−1^ ICG) under very mild NIR irradiation (0.3 W cm^−2^, 2 min). Therefore, introducing a small amount of photosensitizer can significantly reduce the required dose and metal ions needed to be integrated into LDHs. In addition, recent work has also been carried out to enhance the photothermal conversion efficiency of Cu‐LDH by creating surface coordination defects through acid etching.^[^ ^143^ ^]^ As shown in Figure 8d, the surface‐defective d‐Cu‐LDH/ICG (48 °C) can significantly increase the temperature of mouse skin under mild near‐infrared light (808 nm, 0.23 W cm^−1^, 5 min) irradiation, compared to unetched LDH/ICG (40 °C), which is beneficial for enhancing the photothermal effect. It is worth mentioning that d‐Cu‐LDH can degrade in the TME environment to produce Cu^+^, which triggers the Fenton reaction to achieve CDT for cancer. This study successfully coordinated CDT, PTT, and PDT to achieve non‐drug treatment for cancer.

**Figure 9:**
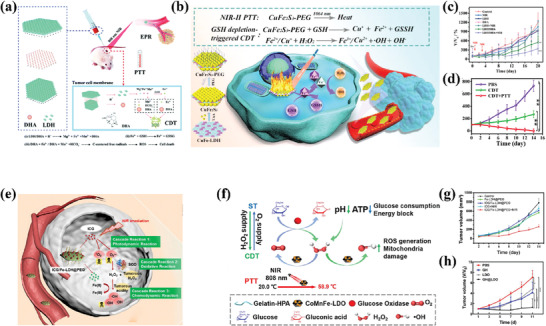
a) Schematic illustration of the DHA delivery system of MnMgFe‐LDH toward efficient loading and mechanism of combined CDT/PTT treatment. Reprinted with the permission from Ref.[138] Copyright 2020, RSC. b) Schematic illustration of the preparation of CuFe_2_S_3_‐PEG NSs for efficient CDT/PTT. Reprinted with the permission from Ref.[144] Copyright 2021, Elsevier. c) Relative tumor volume of mice from the different groups treated under different conditions. Reprinted with the permission from Ref.[138] Copyright 2020, RSC. d) Tumor growth curves of mice with various treatments. Reprinted with the permission from Ref.[144] Copyright 2021, Elsevier. e) Scheme of ICG/Fe‐LDH@PEG mediated catalytic cascade reactions for self‐supplied H_2_O_2_ enhanced CDT. Reprinted with the permission from Ref.[145] Copyright 2022, Elsevier. f) Schematic illustration of the CoMnFe‐LDO composite hydrogel for synergetic tumor chemodynamic/starvation/photothermal therapy. Reprinted with the permission from Ref.[146] Copyright 2022, Elsevier. g) Tumor volume change of 4T_1_ tumor‐bearing mice after different treatments. Reprinted with the permission from Ref.[145] Copyright 2022, Elsevier. h) Tumor volume of tumor‐bearing mice treated with different groups under NIR irradiation. Reprinted with the permission from Ref.[146] Copyright 2022, Elsevier.

PTT has attracted widespread attention due to its non‐invasive nature, precise targeting, reproducibility, and non‐resistance.^[^ ^191^ ^]^ However, its low penetration depth, inadequate photothermal conversion efficiency, and short circulation time can significantly hinder its further application in cancer treatment.^[^ ^192^ ^]^ CDT can convert non‐toxic intracellular substances into toxic substances in the TME through the Fenton reaction, thus providing tumor‐specific treatment and negligible damage to normal tissues.^[^ ^193^ ^]^ However, glutathione (GSH) is overexpressed in the TME and reacts with the generated ·OH, reducing the effectiveness of the Fenton reaction.^[^ ^194^ ^]^ The coordinated use of PTT and CDT is expected to overcome the limitations of monotherapy. As shown in **Figure** **9**a, a recent study constructed a Nano‐drug based on MnMgFe‐LDH to deliver an exogenous chemodynamic reaction trigger, DHA, and achieve PTT and CDT synergistic therapy.^[^ ^138^ ^]^ LDH/DHA degrades in the TME to produce Fe^3+^ and Mn^2+^, which release DHA. The Fe^3+^ produced by LDH/DHA degradation oxidizes GSH to GSSH, thus depleting GSH in the TME. The reduced Fe^2+^ and Mn^2+^ produced by LDH/DHA degradation can undergo chemodynamic reactions with released DHA to generate ·OH, promoting apoptosis of cancer cells. Meanwhile, Mn^2+^ can also generate local toxic heat under NIR irradiation (808 nm), which can improve the efficiency of the Fenton reaction while killing cancer cells. In another study, researchers constructed a PTT/CDT synergistic treatment system based on CuFe_2_S_3_‐PEG (Figure 9b). The similarity between the two works is that they both use the metal ions generated by the constructed LDHs system to consume GSH in the TME to enhance the therapeutic effect of CDT. The difference is that the CuFe_2_S_3_‐PEG system constructed in the latter depends entirely on the metal ions of its own structure to trigger a chemodynamic reaction. Moreover, the PTT of CuFe_2_S_3_‐PEG is achieved under laser irradiation in the second near‐infrared (NIR‐II) window (1064 nm). Compared with the first near‐infrared (NIR‐I) window (700–1000 nm), the penetration depth of NIR‐II is deeper, which can achieve a better PTT therapeutic effect. The photothermal conversion rate of CuFe_2_S_3_‐PEG is 55.86%, significantly higher than that of LDH/DHA (10.7%). A comparison of Figure 9c,d shows that the synergistic PTT/CDT therapeutic effect of CuFe_2_S_3_‐PEG is also better than that of LDH/DHA.

In addition, as the Fenton reaction‐based CDT heavily relies on the concentration level of H_2_O_2_, the efficacy of CDT can be influenced by the endogenous H_2_O_2_ levels in TME.^[^ ^195^ ^]^ To address this issue, some recent works have designed H_2_O_2_ supplementation systems when constructing LDH‐based cancer treatment systems.^[^ ^145^, ^146^ ^]^ As shown in Figure 9e, a recent work designed ICG/Fe‐LDH@PEG NPs containing photosensitizer ICG, which can trigger the photodynamic reaction to produce singlet oxygen (^1^O_2_) and superoxide radical (O_2_^−^) under NIR light irradiation.^[^ ^145^ ^]^ Subsequently, endogenous superoxide dismutase (SOD) can convert O_2_^−^ to H_2_O_2_, greatly increasing the H_2_O_2_ level inside cells. Then, the H_2_O_2_ generated in situ and endogenous H_2_O_2_ in TME can together react with Fe^2+^ produced by the degradation of ICG/Fe‐LDH@PEG NPs through a Fenton reaction, which significantly promotes the production of ·OH, thus enhancing the CDT performance. However, the level of endogenous SOD in cells may also be limited, and the weakly acidic condition (≈ pH 6.5) of TME may also restrict the efficacy of CDT.^[^ ^196^ ^]^ Therefore, in another work,^[^ ^146^ ^]^ researchers designed a Nano‐composite hydrogel (GH@LDO) based on gelatin, nanoenzyme CoMnFe layered double oxide (CoMnFe‐LDO), and natural enzyme glucose oxidase (GOX) was designed for in situ injection therapy of cancer (Figure 9f). The gel ensures the stability of the whole system, and CoMnFe‐LDO as a nanoenzyme can trigger a Fenton reaction to produce ROS for CDT while also generating O_2_. Meanwhile, O_2_ and intracellular glucose can produce gluconic acid and H_2_O_2_ under the catalysis of GOX. Gluconic acid can further lower the pH level in TME, and the generated H_2_O_2_ can supplement the H_2_O_2_ level for CDT, which is beneficial for enhancing the efficacy of CDT. Additionally, glucose based on GOX is also a good starvation therapy (ST), which can lead to insufficient ATP synthesis in cells and cause cell apoptosis. Furthermore, CoMnFe‐LDO also has excellent photothermal conversion efficiency (66.63%). Overall, the nano‐composite hydrogel designed in the study is a multifunctional bioreactor that can synergize CDT/ST/PTT to achieve better cancer treatment. Moreover, GH@LDO is expected to overcome the limitations of CDT efficacy due to insufficient endogenous H_2_O_2_ and the slightly acidic TME environment. In addition, the design based on GOX‐mediated H_2_O_2_ supplementation may be more efficient than the design based on endogenous SOD‐mediated H_2_O_2_ supplementation mentioned in the first study. Therefore, the inhibitory effect of GH@LDO on tumor growth is more prominent than that of ICG/Fe‐LDH@PEG NPs (Figure 9g,h).

#### 2.2.3 Nanotheranostics Platform

Incorporating functional metal ions into LDHs may open a new avenue for developing LDH‐based nano‐diagnostic and therapeutic platforms with advanced functionalities. The physical principle of MRI relies on the amount of water molecules in the region of interest, with the final measurement displaying the spatial distribution of proton relaxation.^[^ ^197^ ^]^ LDHs contain metal ions that are sensitive to MRI, such as Cu^2+^, Gd^3+^, Fe^2+^, Fe^3+^, Mn^2+^, and Co^2+^, which can interact with water molecules and alter the local magnetic field, thus affecting the magnetic resonance.^[^ ^198^ ^]^ Effective MRI contrast agents must accelerate the proton relaxation process to shorten the T_1_ (spin‐lattice)/T_2_ (spin‐spin) relaxation time and result in a higher/lower signal intensity of the target tissue compared to the background signal.^[^ ^199^ ^]^ Based on the different physical properties of nuclear magnetic resonance phenomena, MRI has two imaging modes: T_1_ and T_2_ imaging.^[^ ^200^ ^]^ T_1_ imaging displays high signal intensity for cancerous tissues rich in protein and collagen. While T_2_ imaging performs better than T_1_ imaging for displaying liquid tissues, such as cancerous tissues with edema and necrotic tissues.^[^ ^201^ ^]^

**Figure 10:**
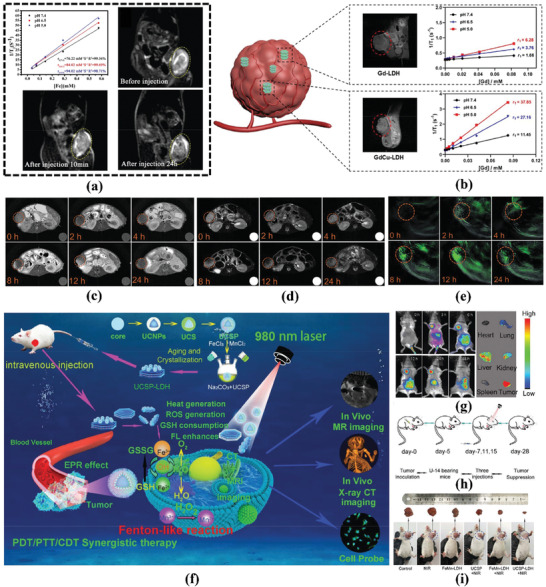
a) Enhanced relaxivity values after incubation with buffer solutions with different pH values at 37 °C for 24 h and MRI images of the 4T1 tumor model after the injection of Fe_200_‐LDH NPs (C(Fe^2+^) = 2.0 mmol^−1^) solution. Reprinted with the permission from Ref.[122] Copyright 2021, RSC. b) Schematic illustration of the in vivo MRI performance of Gd‐LDH and GdCu‐LDH nanoparticles. Reprinted with the permission from Ref.[173] Copyright 2023, RSC. c) In vivo T_1_‐weighted MR images and d) T_2_‐weighted MR images of the tumor‐bearing mice at various time points after i.v. injection of Ce6/CoMn‐LDH nanosheets (tumors are indicated by orange circles). e) PA imaging of the tumor‐bearing mice at various time points after i.v. injection of Ce6/CoMn‐LDH nanosheets. Reprinted with the permission from Ref.[150] Copyright 2020, RSC. f) Schematic illustration for the formation and transport of UCSP‐LDH in blood vessels, EPR‐mediated tumor accumulation, proposed PDT/PTT/CDT mechanism, and multiple imaging functions. g) In vivo fluorescence images of U14‐tumor‐bearing female mice taken after i.v. injection of the UCSP‐LDH nanocatalysts, in vitro fluorescence images of major organs and tumors at 12 h intervals post‐injection with the UCSP‐LDH nanocatalysts. h) Experimentation for the therapeutic model. i) Representative digital photographs of excised tumors in various treating groups of tumor‐bearing mice after 14 days of treatment. Reprinted with the permission from Ref.[147] Copyright 2020, Wiley.

Fe is an essential element in the human body and is widely used as an important functional component in various nanocarriers, with extensive applications in the field of biomedical engineering.^[^ ^202^, ^203^, ^204^ ^]^ In the previous section, it was mentioned that Fe ions can produce photothermal effects for PTT. Additionally, due to the unique paramagnetic properties of Fe, Fe‐based nanomaterials are also widely used as functional components for T_2_‐weighted MRI contrast imaging.^[^ ^205^ ^]^ Recently, an intelligent Fe‐doped LDH/DOX nanoparticle (Fe‐LDH/DOX NP) has been developed as a nanotheranostics platform.^[^ ^122^ ^]^ Fe‐LDH/DOX NPs can accumulate in tumor tissues based on the EPR effect. Then, Fe‐LDH/DOX NPs can degrade and release Fe^2+^ and DOX in the slightly acidic environment of the TME. Under NIR 808 nm laser irradiation (1.5 W cm^−2^, 5 min), Fe^2+^ can generate the photothermal effect (photothermal conversion efficiency 45.67%) for PTT, and the local thermal effect can promote the release of DOX, thereby achieving good synergy between CT and PTT. Moreover, as shown in **Figure** **10**a, the T_2_‐weighted relaxation (r_2_) of Fe_200_‐LDH NP solution increases with decreasing pH, indicating that the TME environment can trigger the MRI effect of Fe^2+^ as a natural endogenous stimulation factor. By observing the T_2_‐weighted MRI images of the 4T1 breast tumor mouse model, it was found that the signal intensity of the T_2_‐weighted MRI images decreased at 10 min and even 24 h after the injection of Fe_200_‐LDH NPs. This decrease in signal can reduce image noise, allowing us to observe the morphology and structure of the tumor more clearly, thus helping us determine the optimal time for tumor treatment.

Gadolinium (Gd^3+^), with high magnetic moments and long electron relaxation time, has become the most extensively studied magnetic metal center in T_1_‐MRI applications.^[^ ^206^ ^]^ In a recent study, researchers for the first time co‐doped Gd^3+^ and paramagnetic Cu^2+^ into LDH to investigate the potential synergistic enhancement of T_1_‐MRI performance by dual paramagnetic metal ions in a nanoparticle.^[^ ^173^ ^]^ As shown in Figure 10b, the MRI performance of GdCu‐LDH exhibits ultra‐high sensitivity to environmental pH, with its longitudinal relaxivity r_1_ increasing as pH decreases. Moreover, at the same pH, the r_1_ relaxivity of GdCu‐LDH is several times higher than that of Gd‐LDH and commercial Gd‐DTPA complexes. Therefore, the results of this study demonstrate the synergistic enhancement of Gd^3+^ and Cu^2+^ in T1‐MRI. Furthermore, due to the high r_1_ relaxivity and pH sensitivity of GdCu‐LDH, it is expected to achieve more effective cancer diagnosis with positive contrast enhancement at doses several times lower than those of commercial T_1_‐MRI contrast agents.

T_1_ and T_2_ imaging in MRI have their respective applications and advantages in cancer diagnosis, and doctors choose appropriate imaging modes for examination and diagnosis based on the specific conditions and types of lesions in patients.^[^ ^207^ ^]^ Meanwhile, MRI imaging can also be combined with other medical imaging techniques such as CTI, PAI, fluorescence imaging, etc., for comprehensive diagnosis, thereby improving the detection rate and diagnostic accuracy of cancer.^[^ ^208^ ^]^ However, traditional single‐mode imaging techniques can only provide local information, which makes it difficult to comprehensively evaluate the characteristics and developmental stages of tumor tissue.^[^ ^209^ ^]^ Notably, multi‐modal imaging materials can combine the advantages of different imaging techniques to obtain information from multiple aspects, thereby improving the accuracy and effectiveness of diagnosis and treatment.^[^ ^210^ ^]^ Therefore, the development of materials with modal functions is of great significance to the field of biomedical imaging.

Recently, research has also focused on the application prospects of LDHs as multi‐modal imaging and therapy platforms. A study has constructed a Ce6/CoMn‐LDH nanodiagnostic and therapeutic platform that enables MRI and PAI dual‐modal imaging and synergistic PDT and CDT.^[^ ^150^ ^]^ Due to the large specific surface area of LDH, Ce6/CoMn‐LDH can achieve ultra‐high loading of photosensitizer Ce6 (93.6 wt.%), which is beneficial to enhance the effectiveness of PDT. The study fully considered the characteristics and limitations of the TME environment. The weak acidic condition in TME can degrade the nanocarrier, while H_2_O_2_ in TME is beneficial to CDT. However, overexpressed GSH in TME can limit the effectiveness of CDT and PDT. Moreover, PDT heavily relies on tissue oxygen to generate singlet oxygen (^1^O_2_), and thus continuous oxygen consumption can worsen tumor hypoxia, leading to inadequate PDT performance. In combination with the above facts, Ce6/CoMn‐LDH can degrade in the slightly acidic environment of TME to produce Co^2+^ and Mn^4+^ and release Ce6. Then, Co^2+^ can undergo a Fenton reaction with abundant H_2_O_2_ in TME to achieve CDT. Mn^4+^ produced by Ce6/CoMn‐LDH degradation can oxidize GSH to GSSG, while Mn^4+^ can also decompose H_2_O_2_ into O_2_. The consumption of GSH and supplementation of O_2_ are beneficial to enhance the effectiveness of CDT and PDT. In addition, Mn^2+^ and Co^2+^ can respectively achieve T_1_ and T_2_‐weighted MRI imaging, making it possible to achieve good MRI imaging (Figure 10c,d). While, based on LDH's optical and acoustic characteristics, Ce6/CoMn‐LDH can also be well suited for photoacoustic imaging (Figure 10e). Moreover, Ce6 is also a good fluorescent dye that can be used for fluorescence imaging. Through comprehensive analysis using different imaging modalities, it was found that the maximum MRI, PAI, and fluorescence signals at the tumor site were observed 12 h after injection of Ce6/CoMn‐LDH. This is helpful for analyzing the circulation cycle of LDH in vivo and formulating appropriate treatment (such as PDT) time. However, it should be noted that to effectively excite Ce6, this study used red light with a wavelength of 650 nm. However, the penetration depth of 650 nm light in tissues is very limited, and it can only penetrate some shallow tissues on the surface of the skin, making it impossible to treat deeper tissues. Therefore, it can be seen that to achieve better therapeutic effects, this study has increased the energy of the laser (15 mW cm^−2^) and the treatment time (30 min), but this may pose potential health and safety risks and hazards.

Additionally, Ce6 was also used as a photosensitizer for PDT, but with the use of mild NIR light (980 nm, 0.75 W cm^−2^, 5 min).^[^ ^147^ ^]^ This study utilized upconversion nanoparticles (UCNPs) doped with rare earth ions, which can convert low‐energy NIR light into high‐energy visible light,^[^ ^211^ ^]^ thus achieving deep tissue PDT of Ce6 efficiently and safely. Specifically, as shown in Figure 10f, UCNPs were coated onto mesoporous silica layers for covalent loading of Ce6 and connected with PEG molecules to enhance biocompatibility (UCSP). Then, the synthesized mesoporous UCSP was anchored onto FeMn‐LDH to obtain UCSP‐LDH. In the slightly acidic microenvironment of TME, UCSP‐LDH would degrade to produce Mn^2+^ and Fe^3+^, which can achieve T_1_ and T_2_‐weighted MRI. Meanwhile, the UCSP anchored in UCSP‐LDH was released in TME, but the complex environment of TME would affect the upconversion luminescence (UCL) intensity of UCSP, which would affect the upconversion efficiency and thus the effectiveness of Ce‐induced PDT. Fortunately, Mn^2+^ and Fe^3+^ ions are effective antioxidants and photodamage agents, which can partially restore the UCL intensity of upconversion materials. Furthermore, FeMn‐LDH can generate photothermal effects under 980 nm irradiation for PTT. Therefore, UCSP‐LDH achieves PTT and PDT simultaneously under the same mild NIR light irradiation. Additionally, the Fe^3+^ and Mn^2+^ generated by the degradation of UCSP‐LDH can undergo a series of reactions in TME, triggering the Fenton reaction to achieve CDT, while also oxidizing and consuming GSH to produce O_2_ in situ to enhance PDT effectiveness. Moreover, Yb^3+^ doped in UCNPs can serve as a CT imaging contrast agent, while Ce6 can also be used for in vivo fluorescence imaging except for being used as a photosensitizer for PDT. As shown in the fluorescence image in Figure 10g, after 12 h of injection of UCSP‐LDH nanocatalysts, they accumulated to the highest level at the tumor site, indicating that 12 h after injection is the optimal treatment time. Therefore, in the subsequent in vivo experiments, the treatment plan of injecting and then using a 980 nm laser (0.72 W cm^−2^, 5 min) to irradiate the tumor site after 12 h was adopted (Figure 10h). The UCSP‐LDH diagnosis and treatment platform constructed in this study can achieve synergistic PDT, PTT, and CDT of cancer under the guidance of CTI, MRI, and fluorescence imaging. As shown in Figure 10i, the tumor size of the experimental mice treated with UCSP‐LDH+NIR was the smallest, demonstrating excellent synergistic treatment effectiveness.

In summary, LDHs can serve as an excellent nanotheranostics platform for cancer mainly due to their excellent biocompatibility, tunable structural properties, and targeted modification capabilities, which enable them to be used for cancer imaging and targeted therapy. Moreover, LDHs are promising candidates for multimodal imaging, which has outstanding application value for comprehensive cancer diagnosis. Therefore, LDHs have significant application potential in the early diagnosis and treatment of cancer.

### 2.3 LDHs for Tissue Engineering

LDHs exhibit excellent biocompatibility and biodegradability, making them highly desirable for various applications in tissue engineering. Notably, MgAl‐LDHs are predominantly utilized in tissue engineering research due to the roles of Mg^2+^ and Al^3+^. Mg^2+^ is an essential trace element in the human body, and its various physiological functions, such as muscle contraction, nerve conduction, and bone metabolism, make it crucial in bone tissue engineering.^[^ ^212^ ^]^ Mg^2+^ has been shown to regulate the differentiation balance of osteoblasts and osteoclasts, facilitating bone tissue regeneration and repair.^[^ ^213^ ^]^ Furthermore, Mg^2+^ exhibits anti‐inflammatory effects and promotes angiogenesis and tissue repair.^[^ ^214^ ^]^ Al^3+^ facilitates cell adhesion and proliferation and enhances scaffold‐tissue compatibility by modulating the biological properties of the extracellular matrix.^[^ ^215^, ^216^ ^]^ The synergistic effects of Mg^2+^ and Al^3+^ in MgAl‐LDHs promote their bioactivity and biocompatibility, thus augmenting their potential for use in tissue engineering applications.

In recent years, the field of tissue engineering has seen a growing interest in the use of LDHs, particularly in the area of bone tissue engineering. Remarkably, the inherent alkaline properties and bioactive components of LDHs confer upon them the ability to modulate the immune response. In a study by Fu et al.,^[^ ^217^ ^]^ CaAl‐LDH calcein nanosheets were developed for the treatment of osteoporosis through a combined approach of acid neutralization and modulation of the immune microenvironment. LDHs underwent degradation in response to acidic conditions due to their mild alkalinity, promoting biomineralization and suppressing osteoclast activity. The presence of Ca^2+^ efficiently induced in situ polarization of bone macrophages toward the M2 phenotype, resulting in the augmentation of regulatory T cells and the suppression of T helper 17 cells. Consequently, this intervention reversed the inflammatory immune microenvironment associated with osteoporosis, thereby promoting bone regeneration. Additionally, LDHs have demonstrated potential as carrier materials, which, when combined with cells and growth factors, can be injected into the body to facilitate the formation of new bone.^[^ ^218^, ^219^, ^220^ ^]^ Moreover, LDHs can be integrated with biocompatible polymers, bioactive molecules, and other materials to develop bone scaffolds with improved biocompatibility and biological activity.^[^ ^221^, ^222^, ^223^ ^]^ Notably, LDH nanosheets can be taken up by osteogenic cells through specific endocytic pathways and can be partially dissolved by acidic organelles or cytoplasm to release bioactive molecules, thereby accelerating osteogenic differentiation.^[^ ^224^ ^]^ Additionally, a recent study has demonstrated the potential of LDHs in nerve tissue engineering through the use of electrospinning technology to fabricate PCL/gel/LDH nanofiber scaffolds.^[^ ^225^ ^]^

**Figure 11:**
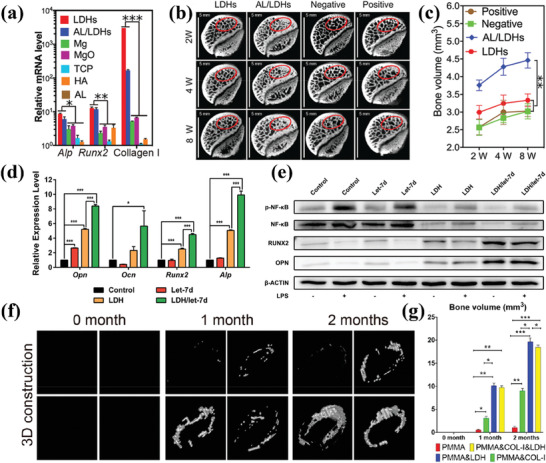
a) Relative gene expression of MC3T3‐E1 cells for osteogenic markers (ALP, Runx2, and Collagen I) on day 7. b,c) Representative cross‐sectional views and quantitative analysis of regenerated bone in the femoral head. The scale bar is 5 mm (b). Reprinted with the permission from Ref.[218] Copyright 2020, RSC. d) Relative mRNA expression levels of osteogenic genes Opn, Ocn, Runx2, and ALP after treatment with let‐7d, LDH, or LDH/let‐7d for 7 days. e) Western blot analysis of RUNX2, OPN, and NF‐κB signaling. Reprinted with the permission from Ref.[219] Copyright 2021, American Chemical Society. f,g) 3D construction of newly formed bone and quantitative analysis. Note: The 3D reconstructed CT images, from left to right and from top to bottom, correspond to the PMMA group, PMMA&COL‐I&LDH group, PMMA&LDH group, and PMMA&COL‐I group, respectively. Reprinted with the permission from Ref.[220] Copyright 2021, American Chemical Society.

Recent developments in materials science have led to the creation of novel bone repair materials that exhibit unprecedented osteogenic differentiation performance. Among these materials are magnesium‐based LDH nanosheets, which have been shown to promote osteogenesis in vitro and in vivo.^[^ ^218^, ^219^, ^220^ ^]^ In a recent study, a Yb‐containing monolayer nanosheet was designed to promote bone regeneration for the treatment of traumatic osteonecrosis of the femoral head.^[^ ^218^ ^]^ Due to the ultra‐high surface area of LDH, the loading capacity and encapsulation efficiency of MgAl‐LDH monolayer nanosheets for alendronate (AL) were as high as 197% and 98.6%, respectively. Runt‐related transcription factor (Runx2), alkaline phosphatase (ALP), and Collagen I are important markers for osteoblast differentiation and bone formation.^[^ ^226^ ^]^ The increase in the expression levels of Runx2, ALP, and Collagen I is an important sign of enhanced osteogenic differentiation ability.^[^ ^227^ ^]^ As shown in **Figure** **11**a, the transcription levels of Runx2, ALP, and Collagen I in mouse embryonic preosteoblasts (MC3T3‐E1) treated with LDHs and AL/LDHs were significantly higher than those in the control group and other common bone biomaterials (Mg, MgO, TCP, and HA), indicating that LDHs and AL/LDHs have excellent in vitro osteogenic differentiation ability. Yb doping facilitated the study of the efficacy of LDHs and AL/LDHs in bone regeneration in vivo by micro‐CT. Injection of LDH or AL/LDH solution into the rabbit femoral head necrosis tunnel not only successfully promoted bone regeneration in the osteonecrosis area but also increased the bone density of the femoral head (Figure 11b), which may be attributed to the therapeutic effect of AL. Furthermore, after eight weeks of treatment, the total bone mass of the rabbit femoral head treated with AL/LDHs was 1.52 times higher than that of the positive control group (autologous bone graft, clinical gold standard) (Figure 11c), indicating that AL/LDHs have a good therapeutic effect on osteonecrosis accompanied by osteoporosis.

miRNA is a potential therapeutic agent, but its delivery remains challenging due to its susceptibility to nuclease degradation and low cellular uptake.^[^ ^228^ ^]^ LDH nanoparticles have also been explored as a delivery platform for miRNA to enhance osteogenic differentiation of mesenchymal stem cells (MSCs).^[^ ^219^ ^]^ MgAl LDH was used as a carrier for miRNA, let‐7d, to synergistically regulate the osteogenic differentiation of MSCs in vitro. As shown in Figure 11d, treatment with LDH or LDH/let‐7d significantly upregulated the expression levels of osteogenic‐related genes. Moreover, LDH and let‐7d exerted a good synergistic effect, promoting MSC differentiation toward the osteoblast lineage, resulting in higher expression levels of osteogenic‐related genes after LDH/let‐7d treatment than with LDH treatment alone. In addition, treatment with LDH and LDH/let‐7d effectively suppressed the LPS‐induced NF‐kB signaling activation (Figure 11e), potentially alleviating inflammatory reactions. Based on the results of this study, LDHs may have dual benefits in reducing inflammation, improving bone regeneration, and repairing inflammatory bone diseases.

Additionally, LDH‐modified bone cement has been developed as a new bone repair material that promotes osseointegration through multiple osteogenic signal pathways.^[^ ^220^ ^]^ Bone cement is mainly composed of polymethyl methacrylate (PMMA) and can be injected into the body to solidify into a solid material, providing support and stability to bone defects, and promoting bone tissue regeneration and repair.^[^ ^229^ ^]^ Typically, PMMA needs to be modified to improve its biocompatibility and biological activity. In a recent study, researchers attempted to modify PMMA bone cement with MgAl‐LDH and mineralized collagen I (COL‐I) either separately or in combination and investigated the effect of modified PMMA on in vivo bone formation. As shown in Figure 11f,g, two months after implantation in rabbit cranial defects, PMMA&LDH and PMMA&COL‐I&LDH treatment groups showed an 18.34‐ and 17.29‐fold increase in bone volume compared to the PMMA group. In addition, PMMA&LDH exhibited better bone‐forming ability than PMMA&COL‐I and PMMA&COL‐I&LDH, indicating that MgAl‐LDH promotes bone formation more effectively in this particular system than COL‐I. Furthermore, the addition of LDH reduced the polymerization temperature of PMMA, which could reduce the thermal damage to bone‐forming cells surrounding LDH‐modified PMMA. Also, the addition of LDH lowered the elastic modulus of PMMA, which is beneficial for reducing stress shielding‐induced bone resorption and indirectly promoting bone integration. However, it should be noted that in this study, the modification of LDH also reduced the mechanical strength of PMMA, which may limit its application by preventing it from providing sufficient load‐bearing support.

Bone tissue engineering has emerged as a promising approach for repairing damaged or diseased bone tissue. In recent years, LDHs have been incorporated into various scaffolds to enhance their biological properties for bone tissue engineering applications.^[^ ^221^, ^222^, ^223^ ^]^ Recently, a study evaluated the effect of a highly porous polycaprolactone scaffold reinforced with LDH on the osteogenic differentiation of bone marrow‐derived mesenchymal stem cells (BMSCs).^[^ ^221^ ^]^ The results showed that the LDH nanoclay reinforced scaffold can promote the osteogenic differentiation of BMSCs, as well as improve the mechanical properties and biocompatibility of the scaffold. In another study, Weng et al.^[^ ^230^ ^]^ engineered an injectable, thermally responsive hydrogel scaffold through the integration of magnesium‐iron LDH into a composite composed of chitosan (CS) and silk fibroin. The resultant hydrogel demonstrated remarkable mechanical characteristics, a reduced gelation timeframe, and a lowered sol–gel transition temperature. Both in vitro and in vivo assessments substantiated the hydrogel scaffold's capacity for fostering angiogenesis and osteogenesis, thereby culminating in the augmentation of bone regeneration. Furthermore, LDHs find applications in the enhancement of scaffold biomaterials. In this regard, Wang et al.^[^ ^231^ ^]^ conducted a study involving the surface modification of porous hydroxyapatite (HAp) scaffolds with magnesium aluminum europium‐LDH (MAE‐LDH) nanosheets. MAE‐LDHs facilitated the controlled release of Mg^2+^ and europium ions, thereby promoting effective bone and vascular regeneration. The incorporation of MAE‐LDHs onto the porous HAp scaffold led to a significant enhancement in specific surface area, surface roughness, and hydrophilicity, thereby providing a greater number of adsorption sites. Consequently, this enhancement led to increased cell adhesion and more efficient osteogenic differentiation. In addition, LDH nanoclay can also regulate the formation of extracellular matrix (ECM), enhance the integration between the scaffold and tissue, and is expected to play an important role in tissue engineering and bone defect repair.

**Figure 12:**
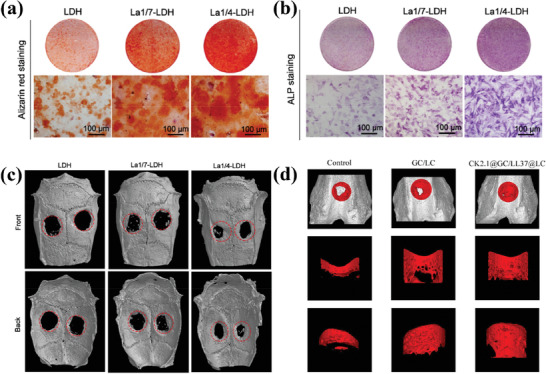
a) Alizarin red staining images and b) ALP staining images of the rBMSCs‐OVX as‐treated with La‐LDH and LDH scaffolds at days 21 and 7, respectively. c) Micro‐CT images of OVX‐rat cranial defects implanted with LDH, La1/7‐LDH, and La1/4‐LDH scaffolds at 12 weeks of post‐implantation. Reprinted with the permission from Ref.[222] Copyright 2021, PMC. d) Micro‐CT reconstruction of repaired tissue of osteochondral defect in vivo. Reprinted with the permission from Ref.[223] Copyright 2021, Elsevier.

CS is a natural biomaterial with excellent biocompatibility and biodegradability.^[^ ^232^ ^]^ Due to its ability to promote osteogenic differentiation and growth of bone cells, thus improving the efficiency of bone tissue regeneration and repair, and has been widely used in bone tissue engineering.^[^ ^233^ ^]^ LDHs have good biocompatibility and biodegradability, and their combination with CS can improve the mechanical properties and biodegradability of chitosan, and also promote the growth of bone cells and regeneration of bone tissue together.^[^ ^234^ ^]^ A recent study has demonstrated the bi‐directional regulation function of lanthanum (La) ‐substituted LDH/CS, which activates osteogenesis while inhibiting osteoclastogenesis, leading to enhanced bone formation and reduced bone resorption.^[^ ^222^ ^]^ La is a member of the rare earth element family and has been identified as a biologically active element that can be utilized for bone disease treatment and bone defect repair.^[^ ^235^ ^]^ In this study, the incorporation of La^3+^ in La‐LDH/CS nanohybrid scaffolds led to the detection of a higher number of calcium nodules in the Alizarin red staining images of La1/7‐LDH and La1/4‐LDH groups compared to the LDH group, as depicted in **Figure** **12**a,b. Additionally, the ALP images exhibited a denser staining pattern in these groups. These observations suggest that the La^3+^ dopant in the La‐LDH/CS nanohybrid scaffold promotes increased ALP activity and ECM mineralization. Remarkably, the La‐LDH/CS scaffold also demonstrated excellent in vivo bone regeneration potential, with the La^3+^ doping facilitating the formation of new bone tissue. Notably, the bone regeneration ability was found to increase with an increase in the amount of La^3+^ dopant, as illustrated in Figure 12c. In another study, researchers designed a biomimetic two‐layered scaffold (CK2.1@GC/LL37@LC) based on peptide‐modified hydrogel/LDH/CS for simultaneous repair of articular cartilage and subchondral bone.^[^ ^223^ ^]^ The upper layer of the scaffold (CK2.1@GC) was composed of a β‐glycerophosphate hydrogel (GC) encapsulated with peptide CK2.1 for upper‐layer cartilage regeneration. The lower layer of the scaffold (LL37@LC) was composed of LDH/CS scaffold (LC) modified with peptide LL37 for subchondral bone regeneration. After 12 weeks of implantation in animal models, micro‐CT analysis showed that in the control group or the group treated with a pure biphasic scaffold (GC/LC), nascent subchondral bone only partially grew in the edge or only the upper level of the defect region (Figure 12d). However, in the peptide‐modified two‐layered scaffold (CK2.1@GC/LL37@LC), the new subchondral bone grew throughout the defect area with fewer gaps. Furthermore, LL37@LC could attract MSCs and promote angiogenesis in vitro. Importantly, this study highlighted the biomimetic layered structure design of the bone scaffold, which could provide a new platform for the integration of new bone tissue and vasculature.

### 2.4 LDHs for Coatings

Compared to traditional biomaterials, magnesium‐based materials have excellent bone‐promoting performance,^[^ ^236^ ^]^ better biodegradability,^[^ ^237^ ^]^ and biomechanical properties,^[^ ^238^ ^]^ making them a candidate material for the next generation of implants. Additionally, magnesium has a density and mechanical properties similar to human bones.^[^ ^239^ ^]^ However, the fast corrosion rate and susceptibility to bacterial infection in physiological environments limit its widespread application as an orthopedic material.^[^ ^240^ ^]^ Alloying is one effective method to improve the corrosion resistance of magnesium‐based implants,^[^ ^241^ ^]^ and the elements in magnesium alloys can exhibit biological characteristics. As we mentioned in 2.3, Mg has positive biological effects, and Zn in magnesium is an antibacterial agent.^[^ ^241^ ^]^ However, merely alloying has limited effects on the corrosion resistance and biological activity of magnesium‐based materials. A common strategy is to further apply coatings to improve the corrosion resistance of magnesium alloys. Coatings commonly used for magnesium alloys include chemical conversion coatings,^[^ ^242^, ^243^ ^]^ polymer coatings,^[^ ^244^, ^245^ ^]^ and micro‐arc oxidation coatings.^[^ ^246^, ^247^ ^]^ However, most of these coatings only provide a physical barrier, which limits their ability to improve the corrosion resistance of magnesium alloys and can also cause biotoxicity issues.

**Figure 13:**
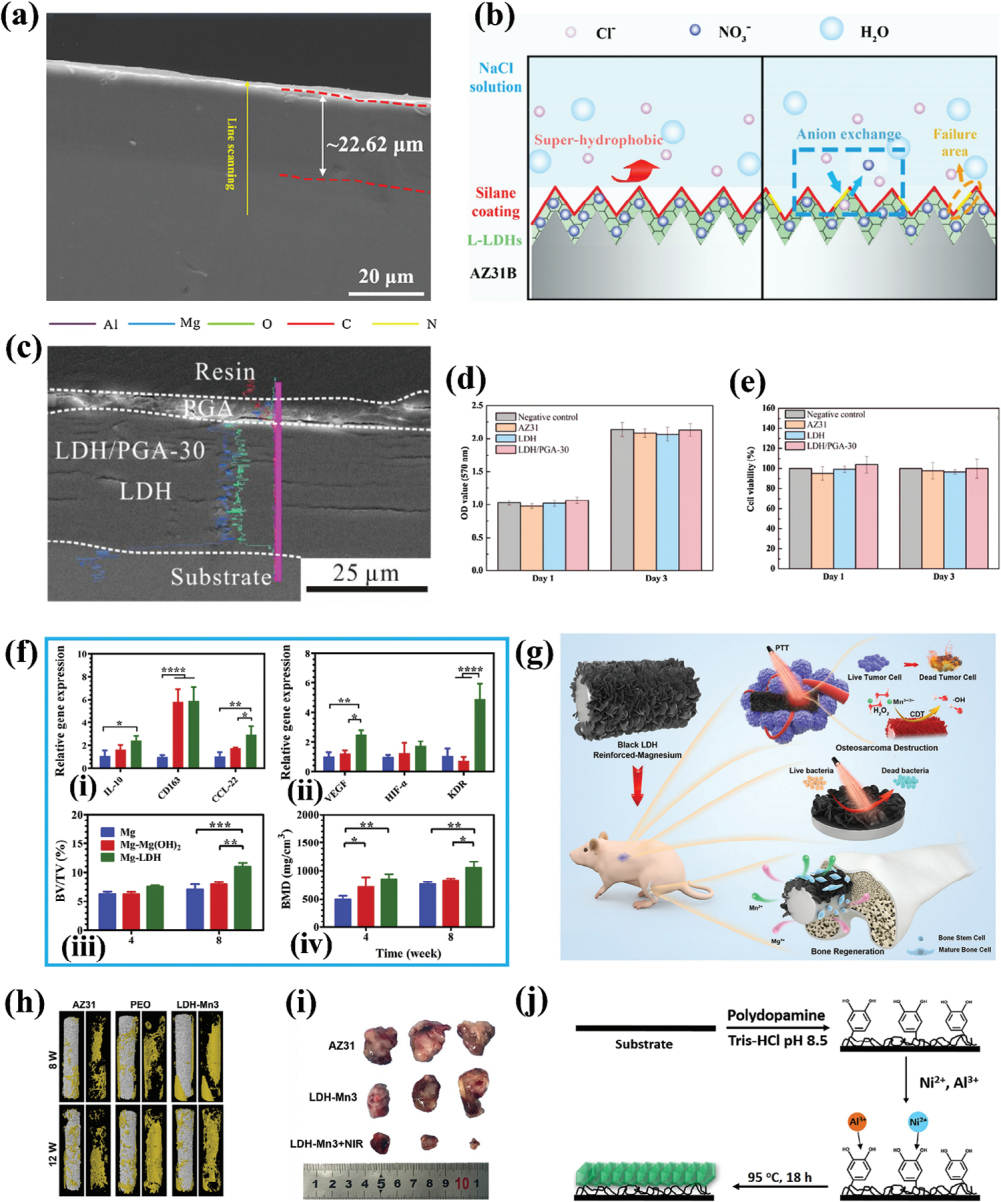
a) Cross‐sectional morphologies of MgAl‐SDS‐LDHs film. Reprinted with the permission from Ref.[248] Copyright 2021, Elsevier. b) Anti‐corrosion mechanism of the composite (L‐LDHs‐OTS) coating. Reprinted with the permission from Ref.[249] Copyright 2021, American Chemical Society. c) Cross‐sectional morphologies and corresponding line scan images (inset) LDH/PGA‐30 coating. d) OD values and e) Cell viability of NIH 3T3 cultured in different extracts prepared with negative control, AZ31 substrate, LDH coated sample, and, LDH/PGA‐30 coated sample for 1 and 3 days. Reprinted with the permission from Ref.[250] Copyright 2020, Elsevier. f) (i)Expression of M2‐related genes of RAW264.7 cultured in the extracts for 3 days. (ii) Angiogenesis‐related gene expression of HUVECs after being cultured in MCM. Calculated bone volume/tissue volume (BV/TV) (iii) and trabecular bone mineral density (BMD) (iv). Reprinted with the permission from Ref.[255] Copyright 2021, Elsevier. g) Stepwise therapeutic strategy for osteosarcoma destruction, antibacterial, and followed bone regeneration. h) 3D reconstruction images of Micro‐CT results of the various samples after implantation in the femur of SD rat for 8 and 12 weeks, yellow section indicates new bone. i) Time‐dependent tumor‐growth curves of the mice after different treatments. Reprinted with the permission from Ref.[251] Copyright 2022, Elsevier. j) Scheme of preparation of NiAl‐LDHs film using PD as a platform. Reprinted with the permission from Ref.[257] Copyright 2017, Elsevier.

Due to the good biocompatibility, unique structure, and ion exchange capability of LDHs, LDHs have been widely studied as potential coatings for magnesium alloys.^[^ ^248^, ^249^, ^250^, ^251^ ^]^ Currently, Mg‐based LDHs have been studied more extensively as coating materials for magnesium alloys.^[^ ^248^, ^249^, ^250^, ^251^ ^]^ Mg‐based LDHs can be synthesized in situ on the surface of magnesium alloys using hydrothermal synthesis or co‐precipitation methods because they contain magnesium as a structural element. Additionally, since Mg‐based LDHs and the magnesium alloy substrate share the metal magnesium, the Mg‐based LDHs coating can achieve dense coverage and tight adhesion on magnesium alloys (**Figure** **13**a). In a recent study, laser etching was used to treat the surface of AZ31 magnesium alloy before the in‐situ generation of MgAl LDHs via the hydrothermal method.^[^ ^249^ ^]^ Laser etching can enhance the hydrophilicity of the magnesium alloy surface, which is conducive to the in‐situ generation and tight adhesion of hydrophilic LDHs. After the in‐situ generation of MgAl LDHs hydrophilic coating on the laser‐etched magnesium alloy surface, further modification of the surface was done with octadecyl‐trimethoxy‐silane (OTS) to form a superhydrophobic coating. As shown in Figure 13b, good super hydrophobicity can reduce the contact area between the composite coating and the corrosive medium, while the ion exchange capacity of the dense LDH layer and the dense Si‐O‐Si network structure inside the OTS layer hinder the penetration of corrosive ions. In addition, when the Mg^2+^ and Al^3+^ accumulated by coating corrosion reach a certain concentration, LDH will recrystallize in the defect area, achieving self‐healing of the LDH coating.^[^ ^252^ ^]^ Typically, LDH coatings consist of a dense internal thick layer and a porous external thin layer.^[^ ^253^, ^254^ ^]^ The porous external layer of LDH coatings can be invaded by corrosive ions, thus reducing the corrosion resistance of the LDH coatings. Therefore, in order to improve the long‐term protection of LDH coatings, a synthetic polypeptide, poly‐L‐glutamic acid (PGA), was used in another study to seal the in‐situ synthesized LDH coating on magnesium alloy surface (Figure 13c).^[^ ^250^ ^]^ It is noteworthy that the LDH/PGA hybrid coating not only improves the corrosion resistance of magnesium alloy but also exhibits good biocompatibility, which provides a certain reference for the safe application of biomedical LDH coatings (Figure 13d,e).

Although the corrosion resistance and biocompatibility of MgAl LDH coatings have been extensively explored, there is still limited research on the effect of MgAl LDH coatings on osteogenic‐related cells and their in vivo performance. Therefore, in a recent study, researchers systematically investigated the effects of MgAl LDH coatings synthesized in situ on pure magnesium on osteogenesis, angiogenesis, and immune response.^[^ ^255^ ^]^ As shown in Figure 13f(i), murine‐derived macrophages (RAW264.7) co‐cultured with Mg‐LDH had higher expression of M2‐related genes, and M2 phenotype macrophages can secrete a large amount of anti‐inflammatory cytokine and growth factors to promote new bone growth.^[^ ^256^ ^]^ Additionally, vascularization‐related genes in human umbilical vein endothelial cells (HUVECs) cultured in macrophage‐conditioned culture medium (MCM) of the Mg‐LDH group were also upregulated (Figure 13f(ii)). Furthermore, after 8 weeks of implantation in mice, implants coated with Mg‐LDH have the highest BT/TV and BMD in the femur (Figure 13(iii),(iv)), indicating their good ability to promote bone formation. Therefore, the results of this study support the potential application value of MgAl LDHs as bone application coating.

Interestingly, a recent study combined the strategy of LDHs for synergistic cancer therapy mentioned in Section 2.2.2 and the technology of LDHs as magnesium‐based biomaterial coatings discussed in this chapter to develop a multifunctional MgMn LDH coating.^[^ ^251^ ^]^ The designed MgMn LDH coating not only possesses corrosion resistance, good biocompatibility, and bone formation‐promoting ability as indicated in the above‐mentioned study, but also can be used for CDT/PTT synergistic therapy of osteosarcoma (Figure 13g). As shown in Figure 13h,i, the coating of LDH‐Mn3 not only has good corrosion resistance and bone formation‐promoting ability but also can significantly inhibit the growth of tumors under NIR light excitation. Meanwhile, the photothermal effect of MgMn LDH can also be used for antibacterial purposes, thereby addressing the issue of bacterial infection in magnesium‐based implants.

Although numerous studies have been done on Mg‐based biomaterials using Mg‐LDHs, it should be noted that biomaterials are not limited to Mg‐based materials. As previously mentioned, the extensive research on the use of Mg‐LDHs for Mg‐based biomaterial coatings is not only due to their unique structural properties and excellent biocompatibility but also because of their ability to achieve in‐situ, tight coating by sharing magnesium with the Mg‐based material. Nevertheless, it also means that applying LDH coatings to other metal‐based or non‐metallic biomaterials presents certain difficulties, limiting the use of LDH coatings as universal biomaterial coatings. Previously, a universal method for LDH coating on any substrate was proposed using a two‐step approach with polydopamine (PD).^[^ ^257^ ^]^ As shown in Figure 13j, a layer of PD coating can be generated on the substrate by oxidation, rearrangement, and polymerization. The abundant catechol groups in the PD layer can serve as surface anchorages for metal ions, allowing metal‐containing LDHs to grow in situ on the PD layer. Therefore, this method is worth considering, as it can expand the application scope of LDH coatings.

### 2.5 LDHs as Functional Membrane

**Figure 14:**
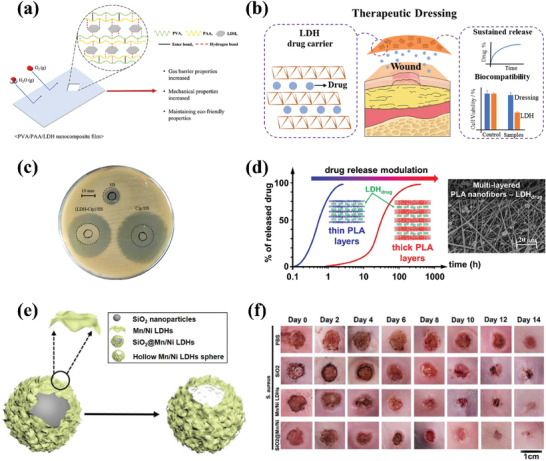
a) Schematic of PVA/PAA/LDH nanocomposite film. Reprinted with permission from Ref.[258] Copyright 2022, Elsevier. b) Schematic illustration of polymer/iron‐based LDH as a wound dressing. Reprinted with the permission from Ref.[261] Copyright 2020, MDPI. c) Photograph of in vitro antimicrobial activity evaluated by Agar disk diffusion assay corresponding to HS, Cip/HS, and (LDH‐Cip)/HS films (6 mm diameter) against Staphylococcus aureus. The dotted line indicates the total area of the swollen films. Reprinted with the permission from Ref.[262] Copyright 2020, Elsevier. d) Schematic tunable of PLA/LDH film with adjustable drug release rate prepared by electrospinning and electrospray technique. Reprinted with the permission from Ref.[263] Copyright 2020, Elsevier. e) The synthetic scheme of the hollow Mn/Ni LDHs structure. f) Photographs of different samples in the mice wound healing model with infection of *S. aureus* after different times. Reprinted with the permission from Ref.[275] Copyright 2020, Wiley.

Recently, there have been studies on the use of LDHs as functional membrane materials, mainly including biocompatible packaging materials^[^ ^258^, ^259^, ^260^ ^]^ and functional wound dressings.^[^ ^261^, ^262^, ^263^ ^]^ Adding LDHs as a filler can effectively enhance the performance of packaging materials. As shown in **Figure** **14**a, LDH blended with polyvinyl alcohol (PVV) and polyacrylic acid (PAA) was used to prepare PVV/PAA/LDH nanocomposite membrane materials.^[^ ^260^ ^]^ As a rigid nanoparticle, LDH can improve the mechanical properties of the composite membrane. Its unique layered structure can also serve as a physical barrier to greatly enhance the gas barrier properties of the packaging material. Furthermore, the biocompatibility of LDHs makes the composite membrane material more advantageous for biomedical applications.

Modern wound dressings are mainly composed of natural polymers or synthetic polymers, such as gelatin,^[^ ^264^ ^]^ pectin,^[^ ^265^ ^]^ polyurethane,^[^ ^266^ ^]^ polyethylene,^[^ ^267^ ^]^ nylon,^[^ ^268^ ^]^ etc. However, conventional dressings only serve as a physical barrier and protection for wounds and do not have bioactivity to promote wound healing. Combining LDHs as drug carriers with dressing materials is expected to develop new types of bioactive wound dressings. As shown in Figure 14b, drug‐loaded LDHs can endow dressings with therapeutic functions. Furthermore, due to the controlled release properties of LDHs and the packaging limitations of dressing materials, drug‐loaded dressings can slowly release therapeutic drugs, thereby prolonging the effective treatment time of dressings and improving their biocompatibility. In a study, researchers blended LDHs loaded with ciprofloxacin (Cip) with hyaluronic acid (HA) to construct a hybrid composite membrane.^[^ ^262^ ^]^ As shown in Figure 14c, the (LDH‐Cip)/HA membrane had significant antibacterial effects. The antibacterial circle was slightly smaller than the Cip/HA group, meaning that LDH, as a drug carrier, makes drug release more controlled, allowing the composite membrane to provide longer antibacterial action, thereby making its bioactivity higher than that of films loaded with pure drugs.

As a bio‐compatible material, PLA has been extensively studied and has potential applications as wound dressings.^[^ ^269^, ^270^, ^271^ ^]^ However, since PLA is hydrophobic, directly embedding hydrophilic drugs in the PLA matrix may result in long‐term uncontrolled drug release due to the incompatibility between the drug and polymer. The composite of drug‐loaded layered double hydroxides (LDHs) with PLA is expected to regulate the drug release from PLA. However, LDHs are hydrophilic and there may be problems with their dispersion in the hydrophobic PLA matrix. To improve the compatibility between LDHs and PLA, many studies have used chemical modification methods to modify LDHs.^[^ ^272^, ^273^, ^274^ ^]^ However, chemical modification may bring about issues with biocompatibility, which limits the biomedical applications of composites. Interestingly, a recent study has used a combination of electrospinning and electrospray techniques to prepare a PLA/LDH composite membrane. Specifically, LDHs were electrosprayed onto the PLA during electrospinning, so that the LDH particles could be evenly distributed on the PLA matrix. Furthermore, by controlling the number of electrospinning layers of PLA and the amount of electrosprayed LDHs, the release rate of the drug‐loaded dressing can be controlled (Figure 14d). This work provides a new approach for us to prepare dressings with specific drug release behavior in the future.

In addition, interesting work has prepared a hollow MnNi LDHs membrane that can be added to dressings for infection prevention.^[^ ^275^ ^]^ Specifically, the MnNi LDHs membrane was first prepared on the colloidal substrate, and then the hollow MnNi LDHs structure was obtained by alkaline etching (Figure 14e). It is worth noting that the metal ions of LDHs can make it act as a peroxidase mimic to trigger the chemical dynamic reaction that converts molecular oxygen into reactive oxygen species, thereby achieving antibacterial effects. The hollow structure of the MnNi LDHs in this study allows it to have better contact with oxygen, thus improving the efficiency of the chemical dynamic reaction and enhancing the bactericidal effect. Furthermore, the hollow LDHs peroxidase has a higher surface area and more active sites, which gives it better bacterial trapping ability and enhances bactericidal efficiency. As shown in Figure 14f, the mouse wound treated with hollow MnNi LDHs exhibited good wound healing after 14 days of treatment, indicating the excellent anti‐infection ability of hollow MnNi LDHs. It is noteworthy that this study did not load any drugs or conduct any modifications toward LDHs. Based on this interesting hollow LDH design, future work on surface modification of hollow drug‐loaded LDHs may further enhance their antibacterial abilities and wound‐healing effects. The method of preparing hollow LDHs based on the colloidal substrate can also inspire us to prepare various shapes of hollow LDH structures, thus possibly endowing LDHs with special properties and more potential applications.

### 2.6 LDH for Biosensors

Biosensors are devices that can convert biological molecules into electronic, photonic, or other signals, enabling high sensitivity, rapid, and accurate detection of these molecules.^[^ ^276^ ^]^ Biosensors have applications in areas such as medical diagnostics,^[^ ^277^ ^]^ life science research,^[^ ^278^, ^279^ ^]^ environmental monitoring,^[^ ^280^ ^]^ and food safety.^[^ ^281^, ^282^ ^]^ Common biosensors are composed of sensing elements, signal transduction elements, and signal processing elements.^[^ ^283^ ^]^ The sensing element is the core component of a biosensor, typically including antibodies, enzymes, DNA, and other molecules.^[^ ^284^ ^]^ However, current biosensors have limitations, including poor selectivity, low sensitivity, poor stability, short lifespan, and high production costs.^[^ ^285^, ^286^ ^]^ In addition, traditional biosensors require complex sample preparation and pre‐processing procedures, which limit their real‐time monitoring capabilities.^[^ ^287^, ^288^ ^]^ LDHs have positively charged plates that can interact with charged biological molecules, such as proteins, DNA, and drug molecules, and selectively capture target molecules based on their specificity, making them promising materials for biosensor research. Compared to traditional biosensors, LDHs biosensors have good selectivity, high sensitivity, good repeatability, and low production costs, and are expected to become a promising type of biosensor.^[^ ^35^ ^]^ In recent years, LDHs biosensors have been primarily studied for the detection of lactate,^[^ ^289^, ^290^ ^]^ DNA,^[^ ^159^ ^]^ H_2_O_2_,^[^ ^292^, ^293^ ^]^ amino acids,^[^ ^294^, ^295^ ^]^ dopamine,^[^ ^294^, ^296^ ^]^ and other biologically active molecules^[^ ^37^, ^297^ ^]^ and drug molecules^[^ ^298^, ^299^ ^]^ in biological samples or living cells. The common strategy for using LDHs in biosensors is to modify the electrode with LDHs‐based materials according to the detection requirements. The commonly used electrode substrates include glassy carbon electrode (GCE),^[^ ^37^, ^291^, ^292^, ^293^, ^294^, ^295^, ^296^ ^]^ screen‐printed electrode (SPE),^[^ ^289^, ^290^, ^298^ ^]^ fluorine‐doped tin oxide (FTO) electrode,^[^ ^299^ ^]^ and modified indium tin oxide (ITO) electrode.^[^ ^297^ ^]^ In recent years, LDHs based on transition metals such as Fe, Mn, Ni, and Co have received great attention because they have outstanding advantages over previous metal catalysts, including high electrocatalytic activity, tunable morphology, increased active surface area, excellent durability, fast electron transfer ability, and high natural abundance.^[^ ^52^ ^]^ Therefore, in recent research related to LDHs biosensors, transition metal‐based LDHs have been widely studied.^[^ ^37^, ^289^, ^290^, ^291^, ^292^, ^293^, ^294^, ^295^, ^298^ ^]^ Here, we have an overview of LDH biosensors and organized the relevant parameters of the LDHs biosensors prepared in these studies in **Table** **3**.

**Table 3:** LDHs biosensors fabricated in recent literature and their related parameters.

Sensing Materials	Substrate	Detection Object	Linearity	Detection Limit	Sensitivity [µA mm ^−1^ cm^−2^]	Reference
NiMn LDH	GCE	H_2_O_2_	16 nm–9.3 mm	2.5 nm	473.07	[292]
FeMn LDH	GCE	Cysteine (CySH) Dopamine (DA)	CySH: 30 nm–9.6 mm DA: 20 nm–700 µm	CySH: 9.6 nm DA: 5.3 nm		[294]
NiCo LDH	SPE	Lactate	5–25 mm	0.533 mm	30.59	[289]
h‐MnNi LDH	GCE	Uracil‐DNA glycosylase		0.48 µm		[37]
h‐NiCo LDH	SPE	Sumatriptan	0.01–435 µm	0.002 µm		[298]
u‐Cu_2_O/CuZn LDH	ITO	S‐nitrosothiols	5 nm–400 µm	1.58 nm		[297]
Au/NiFe LDH	GCE	Scrub Typhus DNA	25 fm–0.5 µm	25 fm		[291]
GO/CoFe_2_O_4_/ZnAl LDH	FTO	Etoposide	0.2–10 µm	0.001 µm	63.408	[299]
MgAl LDH/NiMn_2_O_4_/PANI	GCE	Levodopa	0.1–100 µm	0.005 µm		[296]
CNTs@NiAl LDH	GCE	L‐cysteine	0.05–780 µm	3 nm	351	[295]
ZIF‐67 derived NiCo LDH	SPE	Lactate	0.399 mm	2–26 mm	83.98	[290]
N‐CNTs@NiCo LDH	GCE.	H_2_O_2_	2.5–16987.5 µm	8.72 nm	3998	[293]

**Figure 15:**
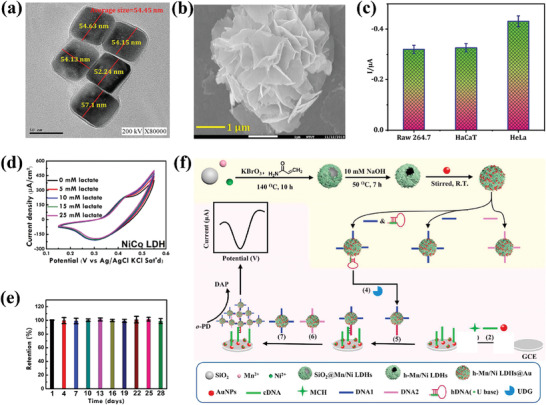
a) TEM images FeMn LDHs‐12h. Reprinted with the permission from Ref.[294] Copyright 2020, RSC. b) FESEM images of NiMn LDH‐180 °C. c) Comparisons of current responses of NiMn LDH toward H_2_O_2_ secreted from Raw 264.7, HaCaT, and HeLa cells. Reprinted with the permission from Ref.[292] Copyright 2021, Elsevier. d) Cyclic voltammograms of the NiCo LDH modified SPE at a scan rate of 50 mV s^−1^ in 0.1 m NaOH with various lactate concentrations (0–25 mm). e) Amperometric response of NiCo LDH modified electrode toward 10 mm lactate for 28 days. Reprinted with the permission from Ref.[289] Copyright 2021, Elsevier. f) Schematic illustration of the proposed signal‐enhanced electrochemical biosensor for UDG detection based on h‐MnNi LDHs. Reprinted with the permission from Ref.[37] Copyright 2021, Elsevier.

It is worth noting that many recent studies on LDHs biosensors have adopted the hydrothermal synthesis method to synthesize LDHs.^[^ ^37^, ^289^, ^291^, ^292^, ^293^, ^294^, ^295^, ^296^, ^299^ ^]^ The morphology of LDHs has a significant impact on their performance as a biosensor, and controlling the morphology of LDHs typically requires optimization of reaction conditions. From the perspective of morphology control, the hydrothermal synthesis method is more suitable for synthesizing LDHs with specific shapes.^[^ ^68^ ^]^ Recently, cubic‐shaped FeMn LDHs were synthesized through hydrothermal synthesis with a reaction time of 12 h for the detection of cysteine in whole blood and dopamine in biological samples.^[^ ^294^ ^]^ As shown in **Figure** **15**a, the average size of the cubic‐shaped FeMn LDHs was 54.45 nm, and they possessed a large surface area and pore size, which helped to improve their electrocatalytic activity and enhance the sensing performance of the biosensor for biomolecules. In another study, researchers prepared flower‐clustered NiMn LDH (Figure 15b) for H_2_O_2_ detection by controlling the temperature of hydrothermal synthesis at 180 °C.^[^ ^292^ ^]^ The ultra‐thin petals of the flower‐clustered structure provided abundant active binding sites for H_2_O_2_, enabling highly sensitive detection of H_2_O_2_. It is worth noting that the NiMn LDH prepared in this study was used for tracking the endogenous H_2_O_2_ produced by live cells. Cancerous HeLa cells can produce more H_2_O_2_ than normal cells due to their rapid oxygen metabolism.^[^ ^292^ ^]^ As shown in Figure 15c, the current response of the NiMn LDH sensor to H_2_O_2_ detected from the HeLa cells was higher than that of non‐cancerous cells, allowing us to distinguish between the two types of cells and potentially aiding in cancer diagnosis. In addition, a similar nanoflower cluster structure biosensor was prepared for lactate detection in sweat but based on NiCo LDH.^[^ ^289^ ^]^ The working mechanism of the NiCo LDH‐based sensor prepared in this study is based on the electrocatalytic oxidation reaction of NiCo LDH toward lactate, which generates an electrical signal on the sensor surface and allows for the detection of lactate concentration. Moreover, the flower‐like NiCo LDH can provide rich electroactive sites for the oxidation reaction of lactate, thus enhancing the oxidation reaction and improving the sensitivity of lactate detection. The lactate concentration in human sweat ranges from 4 to 25 mm.^[^ ^300^ ^]^ As shown in Figure 15d, with the lactate concentration increasing from 5 to 25 mM, the oxidation peak current density of the NiCo LDH‐modified electrode gradually increases, indicating the potential of NiCo LDH as an active material for lactate biosensors. Traditional lactate biosensors are mostly based on enzymes, but enzyme‐based biosensors suffer from stability and sensitivity issues.^[^ ^301^ ^]^ As shown in Figure 15e, the NiCo LDH‐modified electrode prepared in this study exhibits ultra‐stable performance, and the output signal of the amperometric response to 10 mm lactate can still maintain 98.72% of its initial value after 28 days. Due to its good sensitivity and long‐term stability, it provides many possibilities for the application of NiCo LDH as a non‐enzyme lactate biosensor.

In the last part of Section 2.5, we introduced the preparation of hollow LDHs as a type of nanoenzyme for initiating chemical kinetics reactions. The enormous surface area of the hollow LDHs contains numerous active sites, which endow them with the potential to become high‐performance biosensors. Recently, an SPE electrode was modified with hollow NiCo LDHs for the determination of sumatriptan in the presence of naproxen.^[^ ^298^ ^]^ Another interesting study prepared hollow MnNi LDHs (h‐MnNi LDHs) for the detection of uracil‐DNA glycosylase (UDG).^[^ ^37^ ^]^ However, the h‐MnNi LDHs prepared in this study were not directly used to modify the electrode, but were used in a cascade reaction to amplify the low concentration of UDG signal. As shown in Figure 15f, h‐MnNi LDHs were hybridized with hairpin DNA (hDNA), DNA1, and DNA2, respectively, through AuNPs, where hDNA was modified with UDG binding sites. The signal amplification mechanisms of the biosensor prepared in this study utilized the uracil residue generated by UDG as a catalytic activation factor for LDHs. The uracil bases in hDNA can be specifically excised and used to fix h‐MnNi LDHs on the electrode surface through hybridization. Subsequently, low‐concentration UDG signals can be amplified to high‐concentration spectral absorption signals through self‐connection cascade reactions in the form of DNA hybridization, achieving highly sensitive detection of UDG. The hollow structure of the h‐MnNi LDHs deigned in this study provides abundant active sites and a high specific surface area, shortening the length of mass/electron transfer and significantly improving the catalytic activity.

**Figure 16:**
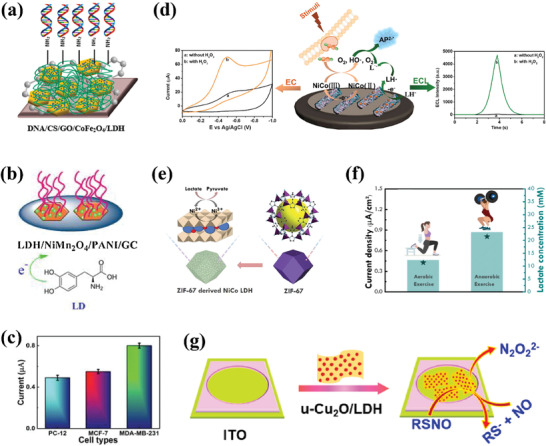
a) Schematic the structure of DNA/GO/CoFe_2_O_4_/ZnAl‐LDH/FTO bioelectrode. Reprinted with the permission from Ref.[299] Copyright 2020, Elsevier. b) Schematic the structure of LDH/NiMn_2_O_4_/PANI/GC bioelectrode. Reprinted with the permission from Ref.[296] Copyright 2020, Elsevier. c) Amperometric current responses of CNTs@NiAl LDH modified electrode sensor for different cell lines. Reprinted with the permission from Ref.[295] Copyright 2022, Elsevier. d) Illustration for the Dual‐Mode Mechanism of Peroxidase‐Like N‐Doped Carbon Nanotubes Loaded with NiCo‐Layered Double‐Hydroxide Nanoflowers for H_2_O_2_ Determination. Reprinted with the permission from Ref.[293] Copyright 2022, American Chemistry Society. e) Schematic illustration of ZIF‐67 derived NiCo LDH for lactate detection. f) Amperometric response of ZIF‐67 derived NiCo LDH modified electrode sensor at applied voltage 0.55 V for different concentrations of lactate. Reprinted with the permission from Ref.[290] Copyright 2022, Elsevier. g) Schematic Illustration of electrochemical detection strategy of synthesized u‐Cu_2_O/LDH for RSNO. Reprinted with the permission from Ref.[297] Copyright 2022, Elsevier.

To enhance the effectiveness and sensitivity of LDH‐based biosensors, researchers frequently adopt the strategy of co‐modifying electrodes with LDHs and other materials.^[^ ^290^, ^293^, ^295^, ^296^, ^299^ ^]^ In one study, NiFe LDH and Au particles were used to co‐modify a GCE electrode for specific detection of scrub typhus DNA.^[^ ^291^ ^]^ Additionally, Fahimeh et al.^[^ ^299^ ^]^ employed CoFe2O4, graphene oxide (GO), and ZnAl LDH to co‐modify the electrode for electrochemical DNA biosensing of drugs (**Figure** **16**a). Furthermore, a ternary nanocomposite of MgAl LDH/NiMn_2_O_4_/polyaniline (PANI) was developed to modify an electrode for human plasma and urine samples (Figure 16b).^[^ ^296^ ^]^ The NiMn_2_O_4_ not only exhibits outstanding catalytic activity but also enhances the conductivity of the electrode material. While, the abundant amine and imine functional groups on the surface of PANI demonstrate a strong affinity for organic molecules, thereby improving the detection efficiency of the biosensor. Moreover, some recent studies used LDHs to wrap carbon nanotubes (CNTs) for electrode modification of biosensors.^[^ ^293^, ^295^ ^]^ CNTs have a high surface area, good conductivity and stability.^[^ ^302^ ^]^ Combining CNTs with LDHs can enhance the biomolecule adsorption of electrodes, thus enhancing the selective adsorption and detection of biomolecules and the application range of LDH biosensors. Recently, NiAl LDH‐wrapped CNTs were prepared for real‐time monitoring of L‐cysteine in cells.^[^ ^295^ ^]^ As shown in Figure 16c, different types of cells express varying levels of L‐cysteine, causing varying degrees of current response on the CNTs@NiAl LDH‐modified electrode, allowing effective discrimination of different cell lines. Moreover, Shen et al. explored a dual‐mode detection strategy based on N‐CNTs@NiCo LDH for H_2_O_2_ secretion in cancer cells to address the poor stability of single‐mode electrochemical biosensors.^[^ ^293^ ^]^ In addition to the electrochemical detection mode, a large amount of solid‐state luminol was loaded into NiCo LDH to achieve electrochemiluminescence detection when luminol was oxidized by H_2_O_2_ (Figure 16d). The flower‐clustered NiCo LDH structure, in combination with N‐doped CNTs and the dual‐mode detection design, enabled the N‐CNTs@NiCo‐LDH modified electrode to achieve ultra‐high sensitivity detection of H_2_O_2_, with a detection limit as low as 8.72 nm.

In addition, two studies have been conducted to construct LDH‐based composite materials through in situ modification methods to modify electrodes, essentially aiming to provide more electrocatalytic active sites to enhance the performance of biosensors.^[^ ^290^, ^297^ ^]^ As shown in Figure 16e, in one study, researchers first constructed an organic framework precursor ZIF‐67 based on cobalt ions and 2‐methylimidazole ligands and then synthesized NiCo LDH in situ on ZIF‐67.^[^ ^290^ ^]^ The ZIF‐67‐derived NiCo LDH has a cage‐like structure, and the multiple valence states of both Co and Ni in the 3D nanocage can serve as solid‐state redox pairs simultaneously, thereby improving the sensitivity of the biosensor. The electrode modified by the ZIF‐67 derived NiCo LDH prepared in this study can achieve enzyme‐free lactate detection and verify the difference in lactate concentration in human sweat under different excise conditions (i.e., aerobic and anaerobic exercise) (Figure 16f). In another study, researchers carried out in situ modification of CuZn LDH to prepare ultrafine Cu_2_O (5 nm) on the LDH sheet structure.^[^ ^297^ ^]^ As shown in Figure 16g, the formed ultrafine Cu_2_O can decompose S‐nitrosocysteine (RSNO) to generate NO, and then the generated NO can be reduced by Zn^2+^ in the CuZn LDH on the electrode while simultaneously converting to an electrical signal, thereby achieving high‐sensitivity detection of RSNO.

## 3 The Clinical Uses of LDHs

### 3.1 Practical Clinical Uses of LDHs

Talcid, a LDHs‐based product commercialized by Bayer,^[^ ^303^ ^]^ has been clinically approved for the treatment of gastrointestinal conditions, such as acidity, gastritis, acidic indigestion, gastroesophageal reflux disease, peptic ulcer, hiatal hernia, and heartburn. Talcid is composed of naturally found MgAl CO_3_ LDH. When exposed to acid in the stomach, MgAl CO_3_ LDH dissociates and releases carbonate, leading to acid neutralization. The amount of acid produced in the intestines is proportional to the dissociation of LDH. Additionally, the pH increase in the stomach deactivates the production of pepsin, which is complexed by Mg and Al cations and adsorbed on the surface of LDH.^[^ ^304^ ^]^ Talcid is available in chewable pastilles (500 mg) or syrup and provides a cheap and effective alternative to other antacid treatments, such as proton pump inhibitors (omeprazole). However, it does not prevent acid production.

Magnesium Iron hydroxycarbonate ([Mg_4_Fe_2_(OH)_12_].CO_3_.4H_2_O), also known as Fermagate, has been investigated as a potential phosphate binding agent in patients with hyperphosphatemia. Hyperphosphatemia, characterized by high levels of phosphates, is commonly observed in patients with chronic kidney failures and can cause calcium imbalances and other related health issues.^[^ ^305^ ^]^ In 2009, a phase II clinical trial on Ferumagate was conducted with a total of 63 hyperphosphatemia patients participating.^[^ ^306^ ^]^ The treatment period was 21 days, and the study drug was administered three times daily before meals. The main endpoint was to reduce serum phosphate levels during this period. In the intention‐to‐treat analysis, the baseline mean serum phosphate level was 2.16 mmol L^−1^. Both the 1‐gram and 2‐gram three‐times daily Ferumagate treatment groups showed statistically significant reductions in mean serum phosphate levels, with averages of 1.71 and 1.47 mmol L^−1^, respectively. The incidence of adverse events (AEs) in the 1‐gram Ferumagate treatment group was similar to that of the placebo group. The 2‐gram group had significantly more AEs, especially gastrointestinal AEs, and more patients stopped treatment due to side effects, making the interpretation of the efficacy of this dose complicated. Both doses resulted in an increase in pre‐dialysis serum magnesium levels. The efficacy and tolerability of Ferumagate were dose‐dependent. In this preliminary phase II study, Ferumagate showed promising efficacy relative to placebo in the treatment of hyperphosphatemia in chronic hemodialysis patients, but the optimal balance of efficacy and tolerability needs to be determined from future dose titration studies or fixed‐dose comparison studies with higher doses. Then, the researchers planned to conduct two Phase III studies to evaluate the effectiveness and safety of fermagate in hemodialysis patients suffering from hyperphosphatemia. These studies aimed to compare fermagate with Sev (NCT00844662) and LC (NCT00841126), which are the leading phosphate binders in the United States and Europe, respectively. Unfortunately, both studies were terminated before reaching their expected completion dates in 2011. Fermagate (Alpharen) was first developed by Ineos Healthcare and its global rights are currently owned by OPKO Health Inc. As stated on the producer's website, fermagate is still in the Phase III development stage for the treatment of hyperphosphatemia in dialysis patients in both the US and Europe. Additional Phase III development was planned for 2014, but the results of these studies have not yet been published.^[^ ^307^ ^]^ Although the clinical trials have not yet been published, Fermagate's potential as a phosphate‐binding agent makes it a promising candidate for future research and development.

### 3.2 Clinical Translation of LDHs: Coexisting Challenges and Opportunities

The goal of biomaterial research is to develop safer and more effective biomaterials to meet the clinical needs of medical treatment and devices.^[^ ^308^, ^309^ ^]^ So far, numerous biomaterials such as polylactic acid,^[^ ^310^ ^]^ collagen,^[^ ^311^, ^312^ ^]^ and natural anticoagulants^[^ ^313^ ^]^ have achieved successful clinical translation and widespread application in the field of biomedical science. Typically, the research and development process of biomaterials can be divided into five stages: basic experimental research, pre‐clinical in vivo evaluation, pre‐clinical research, clinical trials, and post‐market monitoring.^[^ ^314^, ^315^, ^316^, ^317^, ^318^ ^]^ Generally, the entire research and development process of biomaterials is a lengthy process that requires strict safety and efficacy at each stage, and compliance with laws, regulations, and ethical requirements.^[^ ^319^, ^320^, ^321^, ^322^, ^323^, ^324^, ^325^, ^326^, ^327^, ^328^ ^]^ Based on the rigorous process, some biomaterials have been eliminated due to safety issues or insufficient efficacy. For example, Biopure developed a blood substitute product, Hemopure, which was made from other drugs and caused some negative effects, and therefore failed to pass FDA approval.^[^ ^329^ ^]^ Polypropylene mesh was widely used for hernia repair in the 1990s due to its low cost and ease of use.^[^ ^330^ ^]^ However, over time, it was found that the mesh could cause chronic pain and other complications, resulting in numerous lawsuits and product recalls.^[^ ^331^ ^]^ Therefore, before actual clinical translation, biomaterials need to be thoroughly evaluated and tested to ensure their safety and efficacy.

Although LDHs have been widely studied for various biomedical applications, their actual clinical translation is currently very limited and few products can be found that are undergoing clinical trials. In Section 2, we summarized a large amount of LDH‐related research in various biomedical application areas. However, overall, research on LDHs is still in the early stages, from basic experimental research to pre‐clinical in vivo evaluation. In past LDH‐related research, researchers have evaluated the basic characteristics, biocompatibility, toxicity, bioactivity, and in vitro behavior of LDHs. However, the basic experimental research on LDHs has shown many shortcomings and limitations, which have hindered the further development and application of LDHs as biomaterials. In the application process of materials, the preparation and characterization methods are crucial for their performance and application effects.^[^ ^332^, ^333^, ^334^ ^]^ Currently, the preparation and characterization methods of LDH materials have not been standardized, which also leads to some uncertainty in the application process of LDH materials. There are many synthesis methods for LDHs, but the existing methods have some problems, such as the complexity of the synthesis conditions, low yield, poor purity, and uneven particle size, which can affect the properties and applications of LDHs.^[^ ^68^ ^]^

Furthermore, the diversity in LDH synthesis methods and their customizable structural properties have resulted in the synthesis of various LDH types with distinct elemental compositions, shapes, morphologies, thicknesses, and sizes across different research studies. These physical characteristics of LDHs play a pivotal role in shaping their performance within biomedical applications and significantly influence their interactions with biological systems, ultimately impacting their overall effectiveness. For example, the shape and morphology of LDH nanoparticles can exert influence over critical factors such as cellular uptake, biodistribution, and the release kinetics of encapsulated drugs.^[^ ^335^ ^]^ LDH nanosheets, characterized by a high surface area‐to‐volume ratio, may offer enhanced drug‐loading capabilities and efficient cellular internalization.^[^ ^54^ ^]^ Moreover, designing ultrathin LDH nanosheets with just a few layers or as monolayers provides a strategy to optimize cargo anchoring sites, consequently enhancing drug loading capacity.^[^ ^336^ ^]^ Additionally, LDH particle size emerges as a vital determinant affecting biodistribution and cellular uptake.^[^ ^337^ ^]^ Small nanoparticles may exhibit improved cellular internalization but might be swiftly cleared from the bloodstream by the reticuloendothelial system.^[^ ^133^ ^]^ In contrast, larger particles may prolong the circulation time and experience less efficient cellular uptake. Indeed, the versatile structures and compositions of LDHs enable their tailored design for intricate biomedical applications. Nevertheless, this diversity poses challenges in comparing research outcomes across different studies addressing similar issues, often leading to a proliferation of similar yet non‐reproducible and non‐comparable research, which in turn results in significant resource and effort wastage. Moreover, discrepancies in LDHs' structural properties and compositions may introduce variations in their performance, potentially raising safety concerns in the context of their biomedical utilization.

In addition, although many studies have shown that LDHs have good biocompatibility in vitro and animal models, there is still a lack of long‐term biocompatibility and toxicity evaluation of LDHs. As a new type of material, the biological degradation, metabolism, and clearance mechanisms of LDHs in vivo are still unclear, and there are maybe potential toxicity issues. For example, currently, MgAl LDHs are widely used as research objects in biomedical applications. However, aluminum is different from endogenous metals, and its elimination is not regulated and its accumulation in different tissues has been confirmed.^[^ ^338^, ^339^ ^]^ Although, a comprehensive evaluation of the degradation mechanisms of various LDHs has not been conducted to date, Cao et al.^[^ ^62^ ^]^ conducted a visual observation of the degradation kinetics of FeAl‐LDH using real‐time TEM. Their findings revealed an initial decomposition of the nanosheet edges within the first 30 min, followed by a structural collapse of the main LDH framework within 2 h. Simultaneously, a notable influx of H^+^ ions penetrated the interlayer galleries of FeAl‐LDH, leading to the protonation of central OH^−^ groups. This phenomenon hastened the observed nanosheet collapse, underscoring the influence of an acidic environment on the degradation dynamics of LDHs. Moreover, the degradation byproducts of LDHs were found to exhibit a propensity for facile metabolism and excretion through the hepatic and renal pathways.^[^ ^340^, ^341^ ^]^ These byproducts were detectable in both feces and urine samples, thus holding promise for the high biocompatibility and potential biomedical applications of LDHs. However, there is currently a lack of a comprehensive understanding of the specific metabolic pathways and degradation mechanisms of LDHs in vivo. Particularly, with regard to various types of LDHs, they may exhibit distinct characteristics in terms of metabolism and degradation, thus adding complexity to the research. Presently, the monitoring of LDHs' metabolism and degradation in vivo often relies on sampling and analysis, lacking real‐time monitoring methods, which makes it challenging to track the dynamic behavior of LDHs. Furthermore, the majority of studies have focused on short‐term investigations of LDHs' metabolism and degradation. However, for long‐term exposure and potential accumulation effects, further long‐term research is required. Additionally, there is a gap in our understanding of the kinetic behaviors of LDHs in vivo, such as uptake rates, distribution kinetics, and clearance rates, which are crucial for optimizing the biomedical applications of LDHs. In summary, the further advancement and widespread adoption of LDH materials are impeded by several factors, including the absence of standardized preparation and characterization protocols, the need for long‐term biocompatibility and toxicity assessments, and the comprehensive understanding of metabolic pathways, biodistribution, and degradation mechanisms across various LDH variants.

Despite facing many challenges in clinical translation, LDHs, as a new type of nanobiomaterial, possess unique structures and properties and are expected to bring new solutions to the biomedical field. From recent research results summarized in Chapter 2, LDHs have shown enormous potential in various biomedical fields, including drug delivery, cancer diagnosis and treatment, tissue engineering, functional coatings and membrane materials, and biosensors. By controlling the chemical composition, crystal structure, and morphology of LDHs, precise control over their physicochemical properties can be achieved, enabling more accurate applications to meet different needs.^[^ ^342^ ^]^ It is rare to see other biomaterials with the potential to be applied in such diverse biomedical fields as LDHs. Furthermore, with the development of advanced manufacturing technology, the preparation, modification, and functionalization of LDHs are constantly improving, which can provide more possibilities for their applications in the biomedical field. While, the flexibility and modification potential of LDHs structure also create convenience for their multifunctional design, which is expected to address more complex biomedical applications and have great prospects in personalized treatment. In recent years, many studies have focused on the composites of LDHs with other biomaterials, such as graphene,^[^ ^343^ ^]^ carbon nanotubes,^[^ ^344^ ^]^ polymers,^[^ ^345^, ^346^ ^]^ chitosan,^[^ ^73^, ^347^ ^]^ and sodium alginate,^[^ ^348^, ^349^ ^]^ which have the potential to overcome the limitations of single materials, thereby creating greater potential for biomedical applications. Additionally, more and more novel LDH materials have been developed, such as ultrathin LDHs,^[^ ^350^, ^351^ ^]^ monolayer LDHs,^[^ ^341^, ^352^ ^]^ LDHs nanoflowers,^[^ ^353^, ^354^ ^]^ and hollow LDHs,^[^ ^150^ ^]^ which have better physical and chemical properties and broader application prospects. Moreover, in recent LDH‐related research, researchers are increasingly focusing on actual biomedical application environments and problems to promote the substantial clinical translation of LDHs. In recent works, Xu's team developed a PMMA/DMMA coating for LDH surface modification to address the problem of low uptake of LDHs by cancer cells.^[^ ^355^, ^356^ ^]^ The coating can make LDH particles negatively charged in neutral body fluids to prolong blood circulation, and then reverse the charge to positive in tumor tissues to enhance the accumulation and cellular uptake of LDHs. This research achievement is expected to promote effective cancer treatment using LDHs. Simultaneously, researchers are continually dedicated to exploring novel approaches to enhance the targeting capability and therapeutic efficacy of LDH platforms. Recently, Yang and colleagues pioneered the integration of Lactobacillus acidophilus (LA) probiotics with CoCuMo‐LDH nanosheets to engineer photosensitizers responsive to the tumor microenvironment for precise NIR‐II PDT.^[^ ^357^ ^]^ Specifically, LA's inherent hypoxia tropism properties confer exceptional tumor‐targeting capabilities to both LA and CoCuMo LDH. Notably, the low pH (≈5.4) resulting from LA metabolites and GSH transformed the CoCuMo LDH nanosheets into an amorphous state, significantly enhancing the production of ^1^O_2_ under 1270 nm laser irradiation. Moreover, both in vitro and in vivo investigations demonstrated that the LA and CoCuMo LDH therapy effectively triggered apoptosis in cancer cells and resulted in tumor eradication, which provides an excellent strategy for the in situ activation of photosensitizers to achieve accurate NIR‐II PDT. Furthermore, with the continuous development of technology, we can explore the chemical and physical properties of LDHs and their interactions with biological systems more deeply, providing a broader space for the design and optimization of LDHs in biomedical applications. For example, it was found that LDHs can alleviate the embryonic toxicity of chemotherapy drugs by regulating the bone morphogenetic protein‐SMAD signaling pathway.^[^ ^358^ ^]^ This finding helps to understand the mechanism of chemotherapy drug toxicity and provides a theoretical basis for developing new methods to alleviate chemotherapy drug toxicity in the future. Even though LDHs face various challenges in clinical applications, we believe that their prospects in clinical use are still very promising as our understanding of their safety and efficacy deepens. Furthermore, the increasing investment of more and more researchers, funds, and resources, can help accelerate the process of translating LDHs from the laboratory to clinical settings.

## 4 Conclusion and Outlook

This review comprehensively summarizes recent advances in the biomedical applications of LDHs. LDHs have been extensively studied in drug delivery systems, cancer diagnosis and therapy, tissue engineering, coatings, functional membrane materials, and biosensors due to their excellent biocompatibility and outstanding physicochemical properties.

LDHs have potential as drug carriers in delivery systems, but their use alone is insufficient for complex biomedical applications. Recent investigations underscore the significance of functional design tailored to practical application requirements, as well as surface modification to enhance targeted delivery. Encouragingly, the integration of LDHs with magnetic nanoparticles in core–shell structures presents an avenue for efficient targeted delivery controllable through an external magnetic field. Nonetheless, further research is warranted to bridge the gap between concept and practical implementation. Moreover, LDHs have primarily been employed as carriers for anionic drugs due to inherent structural limitations, thereby constraining their application scope. The utilization of LDHs as carriers for non‐anionic drugs remains relatively unexplored, and the existing approaches suffer from drawbacks such as low efficiency and underutilization of the inherent advantages of LDHs. Therefore, future investigations should focus on optimizing LDHs for non‐anionic drug delivery, thereby expanding their application potential. Additionally, it is important to note that despite the considerable body of research on LDHs in the context of delivery systems, our understanding of their fundamental physicochemical properties remains incomplete. Therefore, comprehensive research efforts are warranted to deepen our understanding of LDHs, encompassing their structure, composition, drug loading mechanisms, drug release kinetics, surface properties, and degradation behaviors.

The structural flexibility of LDHs facilitates the development of multifunctional materials for cancer diagnosis and treatment, integrating diverse imaging modalities and novel therapeutic approaches. Although LDH‐based platforms have demonstrated promising outcomes in laboratory and animal studies, their clinical translation encounters challenges. The in vivo behavior and metabolic mechanisms of LDHs remain unclear, and the prevailing targeting strategy relying on the EPR effect exhibits limitations in cellular uptake and targeting efficiency. Moreover, LDH treatment platforms predominantly rely on pH‐responsive release within the tumor microenvironment, lacking flexibility in controlling release rate and dosage. Thus, further investigations are necessary to unravel the biological metabolism and toxicity mechanisms of LDHs, as well as explore precise targeting mechanisms, with the aim of improving imaging accuracy and enhancing the efficacy of coordinated cancer therapy in LDH‐based platforms.

In addition, MgAl LDHs have been widely studied as bone repair materials due to their excellent bone tissue‐inducing and anti‐inflammatory properties. Furthermore, magnesium‐based LDHs can not only protect new biomedical magnesium alloys, but also provide the magnesium alloys with improved biocompatibility, bone‐inducing ability, and other properties. And, LDHs have also been utilized as nanofillers in the development of functional membrane materials, including packaging materials and wound dressings. Moreover, LDH nanoenzymes have garnered considerable attention as biosensors, thanks to their excellent oxidation ability and controllable morphology achieved through hydrothermal synthesis. While LDH biosensors exhibit exceptional features such as high sensitivity and low detection limits, further research is necessary to improve their stability for complex biological sample analysis. In summary, LDHs excel as versatile biomaterials in the biomedical field, offering the flexibility and modifiability of their structure for designing multifunctional LDH‐based biomaterials with expanded potential in the future.

Despite extensive research on LDHs for biomedical applications, their clinical translation remains limited. To bridge this gap, it is crucial to address current shortcomings, establish research standards, and set clear priorities. Optimizing LDHs' preparation methods is essential to improve reproducibility, consistency, and product quality, ensuring their safety and efficacy in clinical applications. Moreover, standardized protocols for preparation and characterization will facilitate results comparison among research works, minimizing redundancy and identifying consistent patterns from a comprehensive database. Additionally, strengthening basic research on LDHs is also necessary to deepen our understanding of their physicochemical properties, providing a robust foundation for practical implementation. Future research should prioritize drug safety, delivery, stability, and efficacy to advance the clinical application of LDHs. Furthermore, establishing a comprehensive clinical trial system is vital to conducting rigorous investigations and validating the effectiveness and safety of LDHs in clinical treatments.

## Conflict of Interest

P.v.R also is a co‐founder, scientific advisor, and shareholder in BiomACS BV, a biomedical‐oriented screening company. The authors declare no other conflict of interest. The authors declare no competing financial interest.
